# A Metalloprotease Homolog Venom Protein From a Parasitoid Wasp Suppresses the Toll Pathway in Host Hemocytes

**DOI:** 10.3389/fimmu.2018.02301

**Published:** 2018-10-23

**Authors:** Zhe Lin, Yang Cheng, Rui-Juan Wang, Jie Du, Olga Volovych, Jian-Cheng Li, Yang Hu, Zi-Yun Lu, Zhiqiang Lu, Zhen Zou

**Affiliations:** ^1^State Key Laboratory of Integrated Management of Pest Insects and Rodents, Institute of Zoology, Chinese Academy of Sciences, Beijing, China; ^2^College of Life Sciences, University of Chinese Academy of Sciences, Beijing, China; ^3^Department of Entomology, College of Plant Protection, Northwest A&F University, Yangling, China; ^4^Institute of Plant Protection of Hebei Academy of Agriculture and Forestry Sciences, Baoding, China

**Keywords:** venom, metalloprotease, toll pathway, *Microplitis mediator*, *Helicoverpa armigera*

## Abstract

Parasitoid wasps depend on a variety of maternal virulence factors to ensure successful parasitism. Encapsulation response carried out by host hemocytes is one of the major host immune responses toward limiting endoparasitoid wasp offspring production. We found that VRF1, a metalloprotease homolog venom protein identified from the endoparasitoid wasp, *Microplitis mediator*, could modulate egg encapsulation in its host, the cotton bollworm, *Helicoverpa armigera*. Here, we show that the VRF1 proenzyme is cleaved after parasitism, and that the C-terminal fragment containing the catalytic domain enters host hemocytes 6 h post-parasitism. Furthermore, using yeast two-hybrid and pull-down assays, VRF1 is shown to interact with the *H. armigera* NF-κB factor, Dorsal. We also show that overexpressed of VRF1 in an *H. armigera* cell line cleaved Dorsal *in vivo*. Taken together, our results have revealed a novel mechanism by which a component of endoparasitoid wasp venom interferes with the Toll signaling pathway in the host hemocytes.

## Introduction

Insects have efficient cellular and humoral immune mechanisms that protect them from invasion by microbes and parasites. Cellular encapsulation of endoparasitoid wasp offspring is an effective and general defense response of hosts (1). Endoparasitoid wasps lay eggs into the hemocoel of their hosts, and their offspring are recognized as invaders and are surrounded by host immunocytes. Multiple layers of hemocytes around the wasp offspring form a capsule, followed by melanization, which eventually leads to killing of the wasps (2, 3). *Drosophila* parasitized by *Leptopilina* has been used as a classical parasitism model to understand the insect immune system. Injection of *Leptopilina* eggs into the host insect triggers the encapsulation processes, including recognition by plasmatocytes and cell-layer formation with further binding of lamellocytes (4–6). Several independent studies established that the Toll/NF-κB pathway of *Drosophila* plays an important role in regulating the cellular encapsulation of parasitoid wasps (7–11). Parasitoid wasps are also used for the biological control of some lepidopteran pests; however, the mechanisms by which wasp offspring suppress the encapsulation response in lepidopteran pest are largely unknown.

The cotton bollworm, *Helicoverpa armigera*, is distributed worldwide and causes significant losses in agricultural produce annually (12). Its natural enemy, *Microplitis mediator*, is a solitary larval endoparasitoid wasp of the family Braconidae and is used as an important biological control agent (13, 14). The parasitoid prefers to lay eggs in the second instar larvae of *H. armigera*, and wasp larvae hatch between 26 and 28 h after oviposition, eventually emerging from the host (15, 16). As a koinobiont parasitoid wasp, *M. mediator* continues to develop within the host after parasitism while the host immune system is suppressed. Based on genomic, transcriptomic, and proteomic studies, it is clear that *H. armigera* has three major immune signaling pathways, Toll, IMD-JNK, and JAK-STAT (17–19). Three NF-κB factors are reported in *H. armigera*, one Relish and two Dorsal proteins. Additional bioinformatics and experimental evidence support the existence of functional Toll and IMD pathways that mediate the induction of antimicrobial peptide (AMPs) via NF-κB transcription factors in *H*. *armigera* (17, 20, 21). Although *H. armigera* immune responses to microbial pathogens are well understood, its responses to macroparasites have not been characterized.

Parasitoid wasps deliver a variety of virulence factors, such as venom proteins, viruses, and virus-like particles into host insects and thereby regulate their immune responses. While most reports are focused on the identification of virulence factors, the molecular mechanism underlying the interaction between virulence factors in wasps and their hosts' immune pathways remains unclear. The bracoviruses (BVs) from the species *Microplitis demolitor*, MdBV, as the major virulence factor is well studied (22–25). The vertically transmitted MdBV integrated its genome segments into the host's genome (26–28). The virions are produced in calyx cells and contain circular double-stranded DNA. During oviposition, wasps inject virions into the body of lepidopteran insects, then the virions immediately infect cells and transcribed virulence genes. Therefore, virulence factors globally suppress the host immunity and development (24, 29–31). Ank-H4 and Ank-N5 are proteins encoded by MdBV of *M. demolitor*. They act as IκB mimics and bind NF-κB factor, thus affecting the IMD signaling pathway and reducing the expression of several AMPs (32). The viral ankyrin (vankyrin) gene, *CsIV-P-vank-1*, from the *Campoletis sonorensis* ichnovirus disrupts the NF-κB signaling pathway similar to its homologs in MdBV (33). To date, there are no reports about venom proteins interfering with NF-κB immune signaling pathways.

Metalloproteases are protease enzymes whose catalytic mechanisms involve divalent cations, usually zinc, in the activation of water molecules. The metal ion is usually held in place by His, Glu, Asp, or Lys amino acid ligands (34). Approximately half of the known metalloproteases have HEXXH pentapeptide as the metal-binding site. Among the 70 families of metalloproteases identified, three classes, M10, M12B, and M13, have been reported in parasitoid wasps. The M10 proteinase contains a pro-peptide, peptidoglycan binding-like domain (Pfam, PF01471), an N-terminal catalytic M10 domain (Pfam, PF00413), and four hemopexin domains (Pfam, PF00045). The M12B proteinase, also referred to as adamalysin or reprolysin, contains a pro-peptide, catalytic M12B domain (Pfam, PF01421) and an additional disintegrin domain. The M12B proteinases have been found in *Pimpla hypochondriaca, Eulophus pennicornis, Chelonus inanitus, M. demolitor*, and other species (23, 35–41). Only the reprolysin-like molecule in *E. pennicornis* venom was reported to exhibit toxicity toward *Lacanobia oleracae* (37). The M13 proteinase consisted of neprilysin-like metalloproteases, which contain M13 proteinase domain (Pfam, PF05649, PF01431). This group has been identified in *Microctonus hyperodae, Aphidius ervi, Leptopilina boulardi*, and *Leptopilina heterotoma* (36, 40, 42). Although metalloprotease has been consistently shown as a component of most parasitoid wasp venom proteins, its function remains largely unknown.

As virulence factors, metalloproteases are involved in a variety of physiological and biochemical processes, including digestion of a wide variety of host proteins, attachment to host cells, cytotoxicity, and compromising the host immune system by decomposing antibacterial peptides (43–46). Specifically, the microbial metalloproteases, such as NleC, AIP56, and PipA, inhibit the function of NF-κB by protein cleavage (47–55). M12B proteinases have been reported in many species as diverse as snakes, spiders, and microbial pathogens (56–60). However, a possible role of parasitoid wasp venom metalloproteases in regulating host immune signaling pathways is not known.

Here, we show that a M12B proteinase from *M. mediator*, which was designated as venom regulatory factor-1 (VRF1), plays a critical role in regulating the egg encapsulation rate in their host. As a parasitic factor, VRF1 is injected into its host, *H. armigera*, after parasitism. Then, the VRF1 proenzyme is cleaved, enabling its entry into host hemocytes at 6 h post-parasitism. In this study, we measured the enzyme activity of recombinant VRF1 and identified its target protein as Dorsal. VRF1 abolished the function of Dorsal, thus preventing transcriptional activation of AMPs. These results reveal a novel mechanism by which a component of endoparasitoid wasp venom disrupts immune-signaling cascades in host hemocytes.

## Methods

### Experimental insects and cell cultures

The colony of *H. armigera* was reared in the laboratory as previously described (17). Beet armyworm, S*podoptera exigua*, and armyworm, *Pseudaletia separata*, were reared under the same conditions as *H. armigera. M*. *mediator*, were maintained at 26 ± 1°C with a photoperiod of 14/10 h (light/dark) and 60 ± 10% humidity in the laboratory. After emergence, adults were provided with 10% honey solution. IOZCAS-Ha-I cell line was obtained from Zhang et al. the Institute of Zoology, Chinese Academy of Sciences (61). Cells were maintained in TNM-FH medium with 10% fetal bovine serum (Sigma).

### Wasp parasitism and egg encapsulation assay

For parasitism experiments, the second instar larvae of *H*. *armigera* were used as hosts. One *M*. *mediator* female was parasitized with a single offspring per host. Then, the hosts were reared individually in 24-wells plates to develop further before dissection at 2, 6, 24, and 48 h post-parasitism. For dsRNA silencing of *M. mediator*, one wasp was used to individually parasitize three hosts. After verifying RNA interference by immunoblot analysis, we continued to count the egg encapsulation rate. For the egg encapsulation assay, the wasp egg or larva was dissected from hosts 48 h post-parasitism under the light microscope. Wasp offspring was divided into three types: larva, partially encapsulated, or complete encapsulated. The egg encapsulation rate (%) was calculated by dividing the number of wasp offspring in each type by the total number of recovered offspring. In each group, 10 depleted wasps individually parasitized 30 hosts (one wasp parasitized three hosts). Each treatment contained five groups. The data from egg encapsulation rate were analyzed using Chi-square test. *T*-tests for statistical analyses (Mann–Whitney nonparametric test) were performed using GraphPad Prism (version 6.0).

### RNA interference

dsRNA was synthesized from specific gene templates according to a previously described method (62). The GFP gene was used as the control. Pupae were selected at 24 h post-pupation for dsRNA injection experiments. A nanoliter 2000 injector (World Precision Instruments) was used to introduce approximately 1 μg dsRNA into the pupal abdomen. Three days later, emerging wasp females were selected for further parasitism experiments. Then, immunoblot analysis was performed on dissected venom reservoirs of individual females to evaluate the efficiency of RNA interference. Venom solution was diluted to 0.2 venom reservoir equivalents (0.2 w.e.) for each sample.

### Rapid amplification of cDNA ends (RACE)

The full-length sequence of *M. mediator VRF1* was obtained by performing rapid amplification of cDNA ends (RACE) using SMART RACE Kit (Clontech). Total RNA was extracted from *M. mediator* venom apparatus. Specific RACE primers were designed based on the cDNA sequences. Primer sequences are listed in Supplementary Table 1.

### Preparation of recombinant VRF1

Bac-to-Bac Baculovirus expression system (Invitrogen) was used to express rVRF1 according to the previously described method (63). Briefly, full-length cDNA encoding VRF1 with the signal peptide was cloned into a pFastBac1 vector. Recombinant pFastBac1-VRF1 plasmids were used to form the bacmid and confirmed by sequencing PCR products. The bacmid DNA was transfected into Sf9 cells using Cellfectin reagent (Invitrogen). As rVRF1 carried 6 × His tag at the C-terminal, rVRF1 was purified using Ni-NTA agarose columns (Qiagen). 2 mg rVRF1 was purified from 2 L conditioned medium. The concentration of purified product was measured using the BCA protein assay kit (Cwbiotech) and stored at −80°C.

### Hemocytes collection and immunofluorescence staining

The hemocytes were bled from second instar *H. armigera* larvae that were unparasitized, 6 h post-parasitism, and 24 h post-parasitism and plated on wells. After 1 h, cells attached on glass slides in Grace's insect cell culture medium (Invitrogen) with phenylthiourea. Fixation, permeabilization, and staining were performed using the Image-iT Fixation/Permeabilization Kit (Molecular Probes) according to the manufacturer's protocol. Phalloidin-Alexa Fluor 594 (Molecular Probes) was used to stain F-actin in the cell cytoskeleton, and Hoechst 33342 (Molecular Probes) was used to stain the DNA. An LSM 710 confocal microscope (Carl Zeiss) was used to visualize the spreading of hemocytes.

### Immunoblot analysis

Whole hemolymph were collected from unparasitized second instar larvae of *H. armigera*, 2 h post-parasitism, 6 h post-parasitism, and 24 h post-parasitism separately. Every sample contained ~10 μL whole hemolymph from 10 second instar larvae of *H. armigera* in a tube containing 1 μL 10 × complete protease inhibitor cocktail (Roche) and phenylthiourea (PTU) (Sigma) on ice. We separated host whole hemolymph into hemolymph (cell free blood) and hemocytes by centrifugation at 1,000 × *g* for 5 min. The hemocytes were washed twice with 1 × PBS. Then, RIPA lysis buffer (Cwbiotech) and 10 × complete protease inhibitor (Roche) were added. The tube was kept on ice for 30 min. Then, it was centrifuged at 12,000 × *g* for 10 min. The supernatants containing cell extracts were collected and stored at −80°C until required. Protein concentration was determined using a bicinchoninic acid protein assay kit (Cwbiotech) and 10 μg of protein from each sample was resolved on a 4–15% gradient SDS-PAGE (Bio-Rad, Hercules, CA, USA) and transferred to PVDF membranes (Invitrogen). The membranes were probed using a primary antibody against *M*. *mediator* VRF1 (1:5,000) or *H*. *armigera* Dorsal (1:5,000), and a secondary horseradish peroxidase (HRP)-conjugated goat anti-rabbit IgG antibody (1:10,000) (Promega). We used *H*. *armigera* (heat shock protein 27.2 kDa (HSP27.2, 1:5,000) antibody against whole hemolymph and hemolymph (cell free blood) samples as a loading control. For the hemocyte samples, we used *H*. *armigera* glyceraldehyde-3-phosphate dehydrogenase (GAPDH) (1:5,000) antibody as the loading control. The polyclonal antibody against *H*. *armigera* Dorsal, *H*. *armigera* HSP27.2, and *H*. *armigera* GAPDH was produced with the full-length purified protein by immunizing rabbits at Beijing Protein Innovation. Immunoblot analysis was visualized using Super Signal West Pico Substrate (Thermo Fisher Scientific).

### Edman sequencing of cleaved rVRF1

To obtain the N-terminal sequence of cleaved rVRF1, the purified protein was digested using trypsin and electrophoresed on SDS-PAGE. The gel was transferred to PVDF membrane and the cleaved product (45 kDa) region of the membrane was excised. Approximately 100 pmol of the collected peptide (45 kDa) was determined using a PPSQ-31A protein sequencer (Shimadzu). Edman degradation was carried out for 6 cycles.

### Enzymatic assay

CEGR (DABCYL-Cys-Glu-Gly-Arg-Ser-Ala-EDANS-NH_2_) produced by GL Biochem (Shanghai, China), was used as a fluorogenic substrate to measure the enzymatic activity. Different amounts of rVRF1 (0, 1, 2, 3, 4, 5, 10, 20, and 40 μM) and 5 μL whole hemolymph from second instar *H. armigera* larvae were incubated with CEGR solution at a final concentration of 0.05 μg/μL. After 30 mins at room temperature, fluorescence was detected using endpoint assay (excitation at 335 nm, emission at 493 nm) on a microplate reader (SpectraMax i3, Molecular Devices). The output data were fitted to the Michaelis–Menten equation using GraphPad Prism (version 6.0).

### Yeast two-hybrid assay

Yeast two-hybrid assay was performed using Matchmaker Gold yeast two-hybrid system (Takara). The full-length coding sequence of VRF1 without signal peptide from the *M*. *mediator* venom apparatus was subcloned into the pGBKT7 (binding domain, BD) vector. The full-length cDNAs of *H*. *armigera Dorsal* were ligated into the pGADT7 (activation domain, AD) vector. Both fusion vectors were transformed into the *Saccharomyces cerevisiae* Y2H Gold strain at the same time. Briefly, colonies containing both vectors were first selected on synthetic defined (SD) medium without leucine and tryptophan (SD-Leu-Trp). Subsequently, they were screened on quadruple-selection SD medium lacking adenine, histidine, leucine, and tryptophan (SD-Ade-His-Leu-Trp).

### Pull-down assay

For pull-down assays, the full-length coding region of *M*. *mediator VRF1* without signal peptide was cloned into pGEX-4T-1vector, and the full-length coding sequence of *H*. *armigera Dorsal* was cloned into pMAL-c5x vector. The tagged proteins were expressed in *Escherichia coli* BL21 cells (Tiangen Biotech). GST-tagged (28 kDa) and GST-VRF1 fusion proteins were respectively bound to glutathione sepharose 4B beads (GE Healthcare). Then, the coupled beads were incubated with MBP-Dorsal fusion protein for 2 h at 4°C. The bound molecules were washed three times at 4°C with PBS buffer. After removing PBS, target proteins were collected with elution buffer (10 mM reduced glutathione in 50 mM Tris-HCl, pH 8.0). The eluted fractions were subjected to SDS-PAGE and followed by immunoblot analysis using anti-GST and anti-MBP monoclonal antibodies (1:5,000; Cwbiotech).

### Transfection of IOZCAS-Ha-I and cell extracts

The VRF1^151−483^ was PCR amplified with venom apparatus cDNA as the template and the primers listed in Supplementary Table 1. SacI (NEB) and SacII (NEB) restriction sites were incorporated into the primers for directional cloning of the gene. PCR-amplified gene fragments were first cloned into PGEM-Teasy (Promega) and then cloned into the expression vector pIZT/V5-His (Invitrogen). IOZCAS-Ha-I cells (61). were maintained in TNM-FH medium (Sigma). Twenty-four hours prior to transfection, cells were seeded at 70–80% confluence in 6-well culture plates (Corning). Two μg plasmids of pIZT/VRF1^151−483^ were diluted per mL of TNM-FH medium without serum followed by adding 8 μL of Cellfectin II (Invitrogen). After 20 min incubation, complete medium was replaced by transfection medium. The transfection medium was then removed after 0, 24, 48, 72, and 96 h and replaced with 1 mL of complete medium. RIPA lysis buffer (Cwbiotech) and 10× complete protease inhibitor (Roche) was added to prepared cell lysate. Protein concentration was determined using a bicinchoninic acid protein assay kit (Cwbiotech). Ten microgram of protein from each sample was resolved on a 4–15% gradient SDS-PAGE (Bio-Rad) and transferred to PVDF membranes (Invitrogen). The membranes were probed using a primary antibody against *M. mediator* VRF1 (1:5,000) or *H. armigera* Dorsal (1:5,000), and a secondary horseradish peroxidase (HRP)-conjugated goat anti-rabbit IgG antibody (1:10,000) (Promega). We used *H. armigera* glyceraldehyde-3-phosphate dehydrogenase (GAPDH) (1:5,000) antibody as the loading control. Immunoblot analysis were visualized using Super Signal West Pico Substrate (Thermo Fisher Scientific).

### Insect injection assay and quantitative real-time RT-PCR

The second instar larva of *H. armigera* was injected with 200 nL of rVRF1 (2 × 10^−7^ μg rVRF1/larva), venom (0.05 w.e. venom/larva), both (2 × 10^−7^ μg rVRF1 and 0.05 w.e. venom/larva) or BSA separately. Then, larvae were challenged with *Beauveria bassiana* (2 × 10^3^ conidia/larva) 24 h latter. Infection experiments of *H. armigera* were performed as described before (21). Each group contained 72 larvae and each experiment was performed in four replicates independently. After 1 day, the hemocytes were collected to extract RNA. Total RNA was extracted using an RNeasy mini kit (Qiagen) and treated with DNase I (Invitrogen). The cDNA was synthesized from 1 μg total RNA using M-MLV Reverse Transcriptase (Promega). Quantitative real-time RT-PCR (qRT-PCR) were performed on a MX3000P system (Stratagene) using a SYBR green PCR Master Mix (Tiangen Biotech). Data were normalized using *Ha Rps3* as the control. The primers used are listed in Supplementary Table 1. Comparative quantification of gene expression was assessed using the ΔΔCt method.

## Results

### VRF1 can modulate the encapsulation rate of *M. mediator* eggs

Our integrative analysis of RNA-seq and proteomic data identified 25 metalloproteases in *M. mediator* venom apparatus, which belongs to M12B and M13 metalloproteases (Supplementary Data 1). To investigate the functions of the metalloproteases, double-stranded RNA of three metalloproteases selected from M12B (MmV189 and MmV94) and M13 (MmV26) families were injected into wasp pupae. To target individual metalloproteases for RNA interference, gene-specific primers (Supplementary Table 1) covering catalytic domain (nucleotides 560-1124 for dsVRF1, 38-543 for dsMmV94, and 1098-1669 for dsMmV26) were designed, and fragments were amplified using PCR. After dsRNA injection, the wasps were used to parasitize *H. armigera* larvae. Rate of cocoon formation was significantly reduced (>50%) in the iMmV189 group, whereas no drastic changes were observed in the iMmV26 and iMmV94 groups (Supplementary Figure 1). Since M12B proteinase, MmV189, can modulate the cocoon formation rate in wasps, we designated it as venom regulatory factor 1 (VRF1).

**Figure 1:**
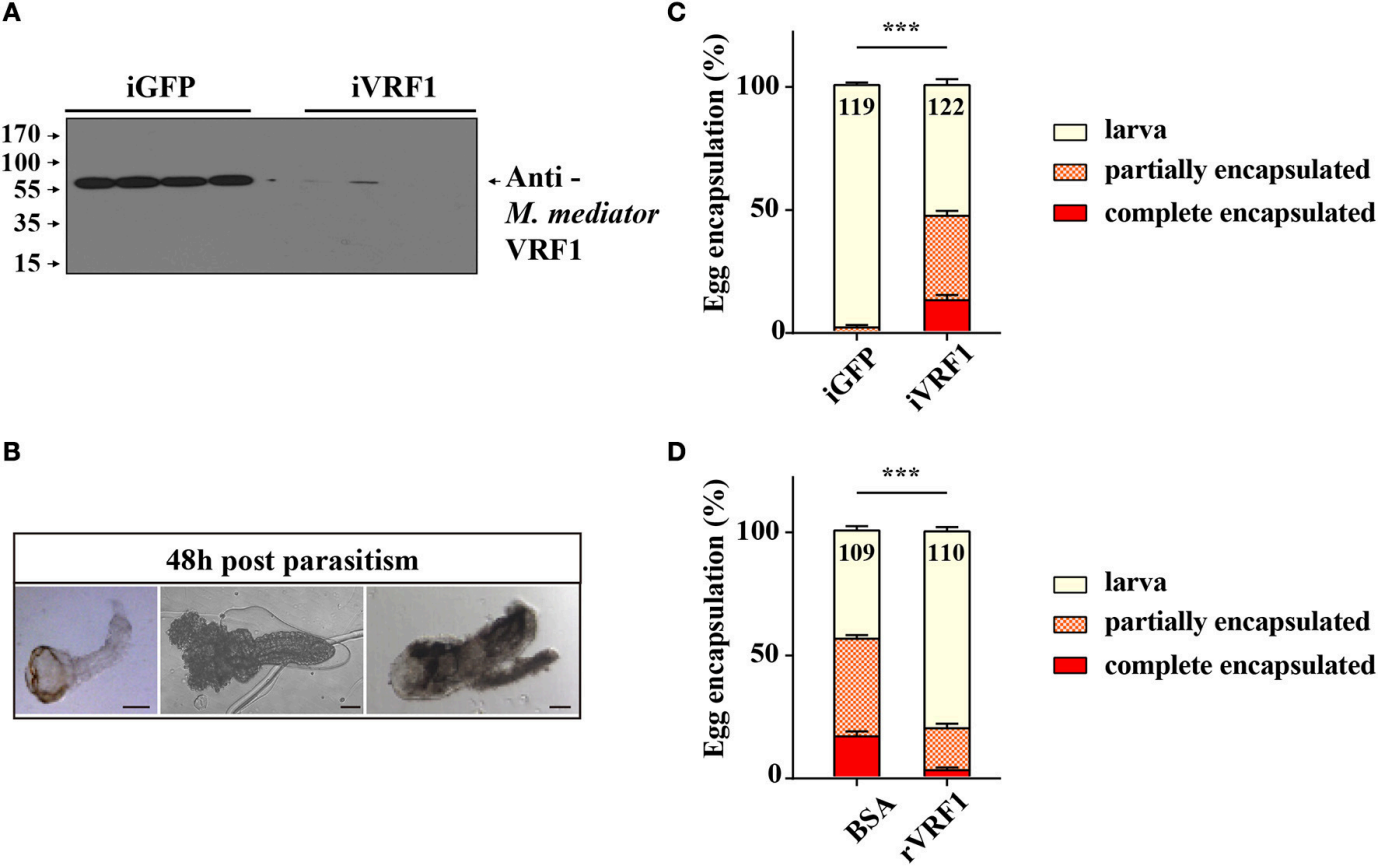
The interaction between *M. mediator* VRF1 and egg encapsulation. **(A)** The efficiency of *M. mediator* VRF1 RNA interference (iVRF1) was confirmed by immunoblot analysis using VRF1 antibody (1:5,000). Three days after injecting dsRNA, venom reservoir was excised from individual females. Venom solution from each sample was diluted to a final concentration of 0.2 venom reservoir equivalents. iGFP injected wasps were used as the control. **(B)** Wasp eggs or larvae were dissected 48 h post-parasitism by visualizing via light microscopy. Wasp offspring were divided into three types based on their phenotype: larva, partially encapsulated, or completely encapsulated (images from left to right). Scale bar, 20 μm. **(C)** Egg encapsulation rate (%) measured after depletion of VRF1 (iVRF1) when compared with dsGFP-injected wasps (iGFP). Each group contained 10 RNAi wasps that individually parasitized 30 s instar *H*. *armigera* larvae. Each RNAi treatment contained five groups. Data represent the numbers of recovered offspring analyzed. Statistical significance of the egg encapsulation rate was analyzed using the Chi-square test. Error bars represent the standard error of the mean (SEM). Statistically significant differences between groups were evaluated by *t*-test (Mann-Whitney nonparametric test), ^***^p < 0.001. **(D)** Egg encapsulation rate (%) was partially rescued following rVRF1 injection. rVRF1, second instar larvae of *H*. *armigera* were injected rVRF1 (2 × 10^−7^ μg rVRF1/larva), 24 h later, were parasitized by VRF1 depleted wasps. BSA, second instar larvae of *H*. *armigera* were injected the same amount of 200 nL BSA, 24 h latter, were parasitized by VRF1 depleted wasps. Each group consisted of 10 RNAi injected wasps that individually parasitized 30 hosts (one wasp individually parasitized three hosts). Each treatment contained five groups. Data represent the numbers of recovered offspring analyzed Statistical significance of the egg encapsulation rate was analyzed using the Chi-square test. Error bars represent the standard error of the mean (SEM). Statistically significant differences between groups were evaluated by *t*-test (Mann-Whitney nonparametric test), ^***^p < 0.001.

For endoparasitoid wasps, the encapsulation response carried out by hemocytes is the major defense mounted by the hosts. To investigate the functions of VRF1, dsVRF1 were injected into wasp pupae followed by immunoblot analysis of dissected venom reservoirs of individual females. We found that the efficiency of RNA interference in venom reservoir was high (Figure 1A). The egg encapsulation rate of dsVRF1 injected wasps was evaluated by recovering wasp offspring from hosts 48 h post-parasitism. As expected, almost all offspring (98.46 ± 0.95%) recovered from hosts parasitized by double stranded green fluorescence protein (dsGFP)-treated wasps, developed into normal larvae (Figures 1B,C). However, the rate of egg encapsulation of dsVRF1 injected wasps was significantly increased, only half (53.13 ± 2.34%) of the *M. mediator* grew into normal larva. About 34.32 ± 1.98% of hatched wasps were partially encapsulated (Figure 1C). Moreover, 12.55 ± 2.19% of wasps were covered with a thick layer of hemocytes and were completely encapsulated (Figure 1C).

We cloned the full-length *VRF1* cDNA from the venom apparatus of *M. mediator* by performing RACE. In order to study the function of *M. mediator* VRF1, we used baculovirus expression system to express VRF1 protein (Supplementary Figure 2). Immunoblot analysis showed that the recombinant protein migrated as a single band of 65 kDa, which is larger than the predicted molecular weight (56 kDa) and could be attributed to glycosylation. Then, we purified the recombinant protein, rVRF1, on a Ni-NTA agarose column (Supplementary Figure 2).

To further determine whether rVRF1 can modulate the encapsulation rate of *M. mediator* eggs, we conducted rescue experiments by injecting rVRF1 protein (2 × 10^−7^ μg rVRF1/larva) or the same concentration of bovine serum albumin (BSA) into the second instar larvae of *H. armigera*, then dsVRF1 injected wasps were used to parasitize the treated larvae of *H. armigera*. In the group injected with BSA, the egg encapsulation rate was 39.60 ± 1.49% for the partially encapsulated type, and 16.37 ± 2.03% for the completely encapsulated type. In contrast, injection of rVRF1 group significantly decreased the egg encapsulation rate; it was 17.38 ± 1.89% for the partially encapsulated type, and 2.61 ± 1.07% for the completely encapsulated type. Accordingly, compared to the BSA group (44.03 ± 1.71%) the percentage of wasp offspring that developed into normal larval stage was significantly elevated in the rVRF1 group (80.01 ± 1.75%) (Figure 1D). This suggested that *M. mediator* VRF1 is an important parasitic factor and that depletion of VRF1 in *M. mediator* significantly increased the percentage of egg encapsulation rate. However, the phenotype was rescued after rVRF1 was injected into the host body.

### VRF1 enters *H. armigera* hemocytes at 6 h post-parasitism

**Figure 2:**
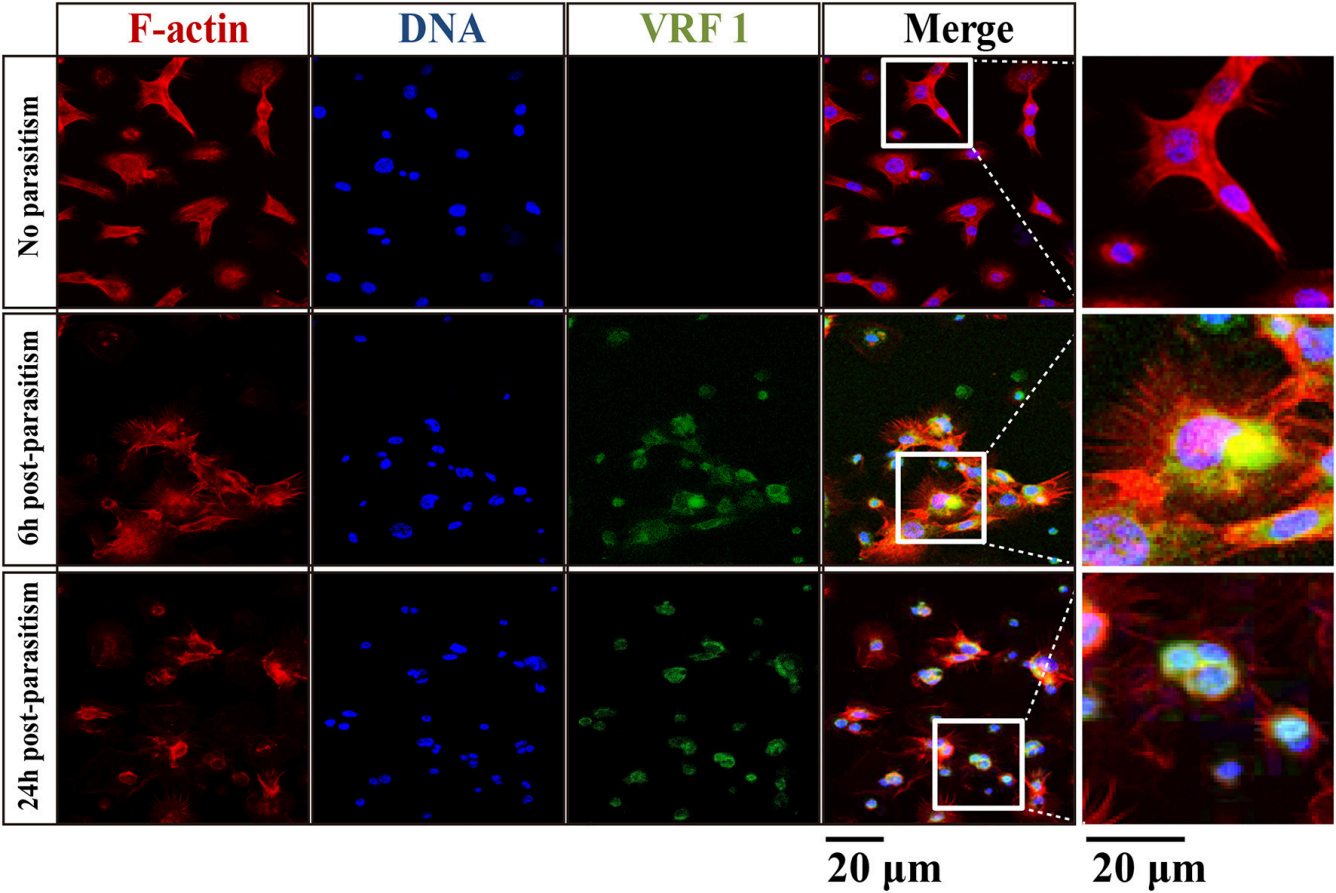
VRF1 localized near host hemocyte nucleus after parasitism. Confocal micrographs of hemocytes from second instar *H. armigera* larvae, no parasitism, 6 h post-parasitism, and 24 h post-parasitism. F-actin and hemocytes DNA were visualized with Phalloidin-Alexa Fluor 594 (red) and Hoechst 33342 (blue) using Image-IT cell labeling kit. VRF1 was detected using a specific rabbit polyclonal antibody (green). The white boxed areas are enlarged in the right panel and show the hemocytes cluster. Scale bar, 20 μm.

In order to verify that VRF1 was delivered into the host's hemocoel after parasitism, second instar *H*. *armigera* larvae were parasitized. Then, cellular location of VRF1 was visualized by immunofluorescence microscopy using the VRF1 antibody. In most hemocytes, the protein was easily visualized as green spots at 6 and 24 h post-parasitism, whereas VRF1 was not detected in hemocytes of unparasitized hosts (Figure 2). Moreover, *M*. *mediator* VRF1 was visualized as green fluorescence close to the nucleus in the hemocytes at 6 h post-parasitism. At 24 h post-parasitism, VRF1 surrounded the host's nucleus. During this time, filamentous actin (F-actin), an essential component of the cytoskeleton, was strongly disrupted.

**Figure 3:**
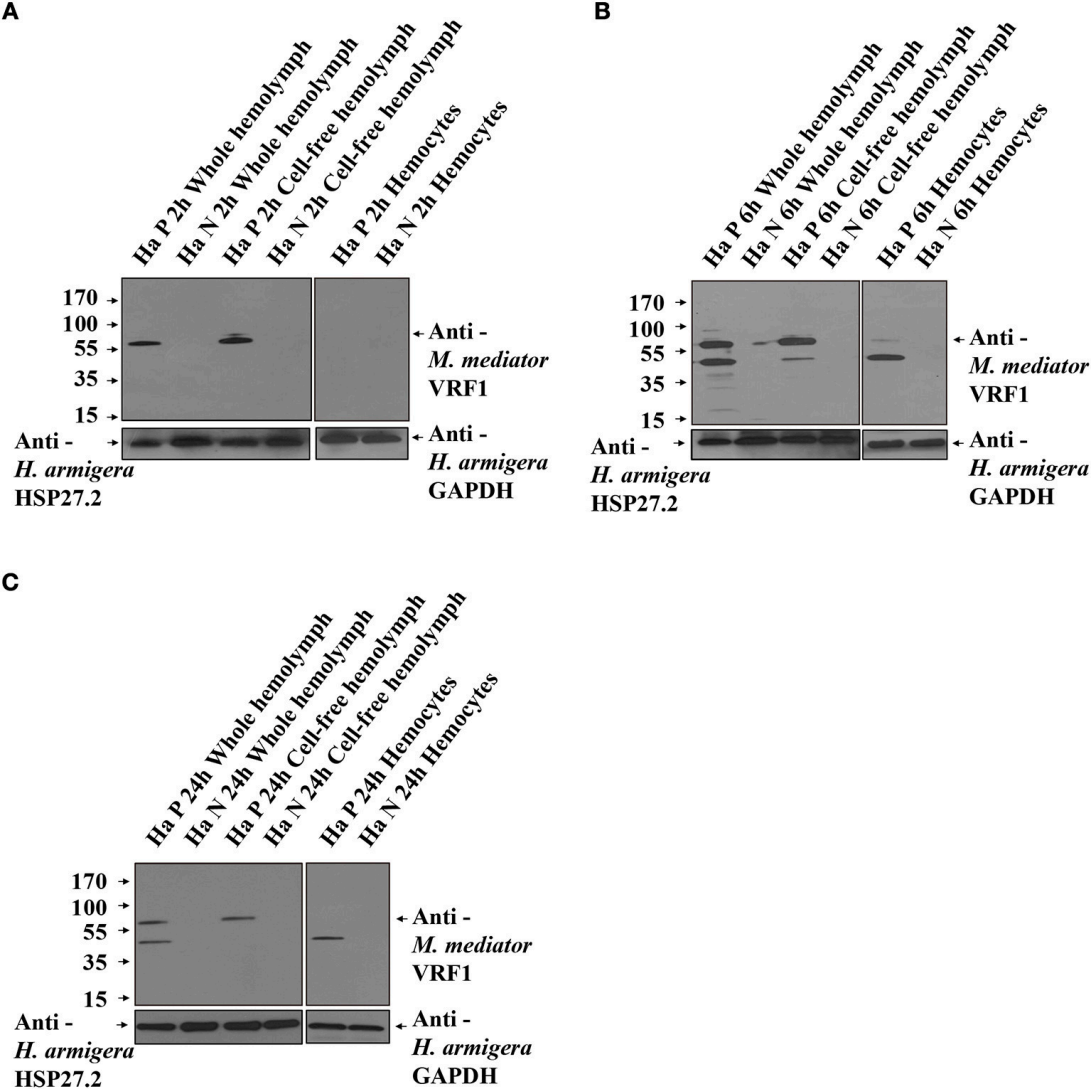
VRF1 was cleaved into two parts after entering natural host's hemocoel. **(A)** Immunoblot analysis of whole hemolymph, cell-free hemolymph, and hemocytes from second instar *H. armigera* larvae at 2 h post-parasitism (P 2h) and unparasitized second instar *H. armigera* larvae (N 2h), using VRF1 antibody. **(B)** Immunoblot analysis of whole hemolymph, cell-free hemolymph and hemocytes from second instar *H. armigera* larvae 6 h post-parasitism (P 6h) and unparasitized second instar *H. armigera* larvae (N 6h), using VRF1 antibody. **(C)** Immunoblot analysis of whole hemolymph, cell-free hemolymph, and hemocytes from second instar *H. armigera* larvae at 24 h post-parasitism (P 24h) and unparasitized second instar *H. armigera* larvae (N 24h), using VRF1 antibody. HSP27.2 (*H. armigera* heat shock protein 27.2 kDa) antibody (1:5,000) was used as the loading control for the whole and cell free hemolymph samples. GAPDH (*H. armigera* glyceraldehyde-3-phosphate dehydrogenase) antibody (1:5,000) was used as the loading control for hemocytes.

To further prove that *M*. *mediator* VRF1 enters into the host hemocytes, we analyzed the hemolymph of parasitized and unparasitized second instar larvae of *H. armigera* using immunoblot analysis (Figure 3A). At 2 h post-parasitism (P 2h), *M*. *mediator* VRF1 protein appeared as a single band at 65 kDa in total proteins isolated from the whole hemolymph and cell-free hemolymph, however, no band was observed in the hemocytes. In the control group (unparasitized *H. armigera* at 2 h), there was no protein band in the corresponding position in the whole hemolymph, cell-free hemolymph, or hemocytes (Figure 3A). At 6 h post-parasitism (P 6h), the ~65 kDa band and an additional ~45 kDa band were detected in the whole hemolymph. While the ~65 kDa band also appeared in the cell-free hemolymph and the ~45 kDa band appeared in the hemocytes (Figure 3B). At 24 h post-parasitism, both proteins were present but became fainter (Figure 3C). At 48 h post-parasitism, the proteins could not be detected. These results indicated that VRF1 proenzyme is cleaved 6 h post-parasitism and enters the host hemocytes in less than 24 h post-parasitism.

To determine whether the cleavage site of *M. mediator* VRF1 was specific, we used *M. mediator* parasitized the other two insect species. *S. exigua*, is an unsuitable host of *M. mediator*, while *P. separata*, is a suitable host. Immunoblot analysis indicated that *M. mediator* VRF1 was not cleaved in hemolymph of *S. exigua* and *P. separata* at 6 h post-parasitism (Supplementary Figure 3), suggesting that the cleavage could be acted by the *H. armigera* hemolymph factor.

### Determination of the rVRF1 proenzyme cleavage site

**Figure 4:**
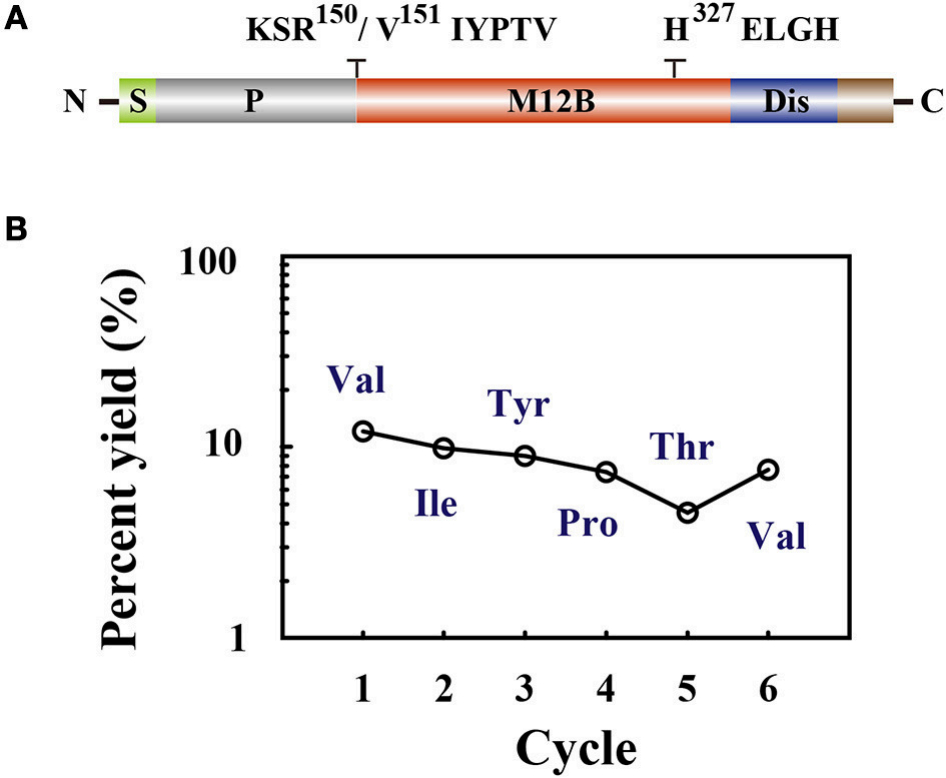
**(A)** The domain structure is indicated as signal peptide (S), prodomain (P), metalloprotease 12B domain (M12B), and disintegrin-like domain (Dis). Amino acid sequences KSR^150^/V^151^IYPTV showed that cleavage occurred after R^150^. The conserved zinc-metalloprotease signature sequences H^327^ELGH shown in their relative positions. **(B)** Amino acid sequences of the collected peptides (45 kDa) were determined by Edman degradation, which was carried out for 6 cycles. The peptide sequence revealed was Val-Ile-Tyr-Pro-Thr-Val.

Members of the M12B family are commonly produced as proenzymes and removal of the prodomain is required to generate the active form (64). The N terminus of *M*. *mediator* VRF1 contains a prodomain, which keeps the proenzyme in an inactive form. As a M12B proteinase, VRF1 contains a conserved M12B catalytic domain (M12B) with two histidine (H^327^ELGH^331^) motifs located in a zinc-binding domain, and a disintegrin-like domain (Dis) (Figure 4A).

In order to identify the cleavage site, limited proteolysis experiments were performed. We incubated rVRF1 with hemolymph from second instar larvae and chymotrypsin, or trypsin. Immunoblot analysis with anti-VRF1 antibody indicated that rVRF1 was cleaved into two fragments of 65 and 45 kDa in the hemolymph and chymotrypsin digested groups. But, in the trypsin digested group, only the 45 kDa protein band was visible (Supplementary Figure 4). Then, the membrane was stripped with stripping buffer and re-blotted with anti-His antibody. In the hemolymph and trypsin digested groups, the protein bands appeared in the same positions. However, both 65 and 45 kDa bands disappeared in the chymotrypsin digested group with anti-His antibody (Supplementary Figure 4B). This suggested that rVRF1 is digested in the hemolymph and that the cleavage site is in the N-terminus.

To determine the cleavage site of rVRF1, we performed Edman sequencing of the 45 kDa fragment from trypsin cleaved rVRF1. The N-terminal amino acid sequence of the first six residues were determined as Val-Ile-Tyr-Pro-Thr-Val (Figure 4B). This result showed that *M*. *mediator* VRF1 as a proenzyme is cleaved between Arg^150^ and Val^151^ peptides to produce a mature protein, and that the C-terminal fragment contains the catalytic domain (Figure 4A).

### rVRF1 can cleave substrates *in vitro*

**Figure 5:**
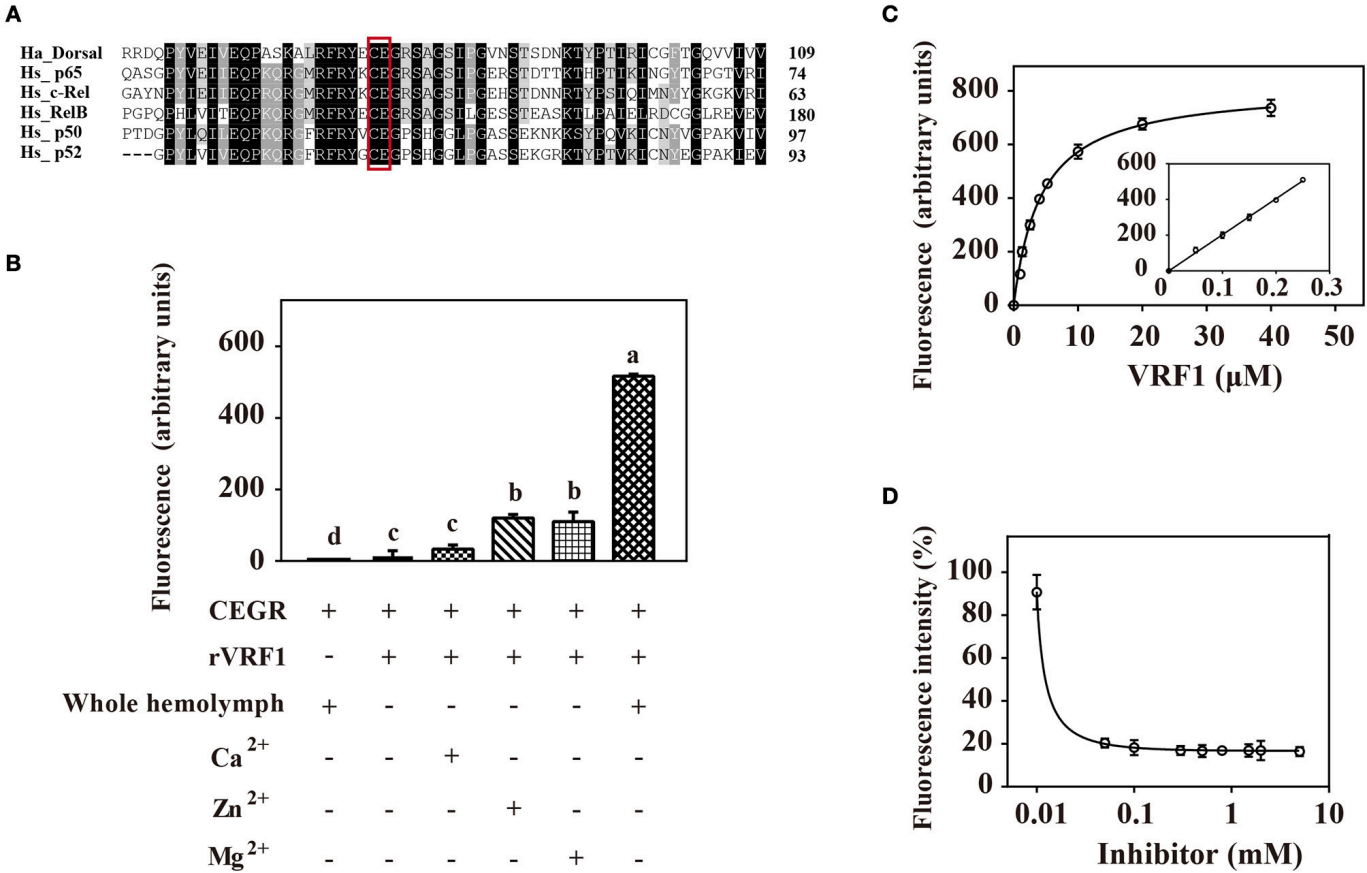
rVRF1 could hydrolyze artificial substrate containing the putative cleavage site. **(A)** Sequence alignment of *H. armigera* Dorsal and human Rel family proteins. Identical or similar residues are highlighted in black and gray colors respectively. Boxed sequences indicate the conserved Cys-Glu residues. **(B)** Fluorescence assay of rVRF1 in different reactions. To test the enzymatic activity of rVRF1, the fluorogenic substrate peptides comprising the sequence DABCYL-Cys-Glu-Gly-Arg-Ser-Ala-EDANS-NH2 (CEGR) were synthesized. Substrate CEGR at 0.05 μg/μL was incubated with rVRF1 at 5 μM at room temperature for 30 min. The final reaction volume of 200 μL contained 50 mM Tri-HCl, 150 mM NaCl, 0.2 mM NaN_3_ with 5 μL whole hemolymph from second instar *H. armigera* larvae or 5 mM CaCl_2_, ZnCl_2_, and MgCl_2_ respectively. Fluorescence was measured using a microplate reader (SpectraMax i3, Molecular Devices) with endpoint assay set at excitation wavelengths of 335 nm and emission wavelengths of 493 nm. Error bars represent the means ± SEM from three replicates. Different letters above a given bar represents significant difference in Tukey's multiple comparison test (*p* < 0.05). **(C)** rVRF1 enzymatic activity assay. Reactions contained 0.05 μg/μL substrate CEGR (DABCYL-Cys-Glu-Gly-Arg-Ser-Ala-EDANS-NH2), the indicated amount of rVRF1 (0, 1, 2, 3, 4, 5, 10, 20, and 40 μM) and 5 μL whole hemolymph from second instar *H. armigera* larvae. After incubation for 30 mins at room temperature, fluorescence was measured using a microplate reader (SpectraMax i3, Molecular Devices) with endpoint assay set for excitation at 335 nm and emission detection at 493 nm. The inset shows an enlargement of the plot depicting the values for low concentrations of rVRF1. Data were fitted to the Michaelis-Menten equation using GraphPad Prism (version 6.0). **(D)** Assay of rVRF1 inhibition curve by 1,10-phenanthroline. Substrate CEGR (DABCYL-Cys-Glu-Gly-Arg-Ser-Ala-EDANS-NH2) at 0.05 μg/μL, 5 μM rVRF1, 5 μL whole hemolymph of second instar *H. armigera* larvae and increasing amounts of 1,10-phenanthroline (Sigma) at 0.01, 0.05, 0.1, 0.3, 0.5, 0.8, 1.5, 2, 5 mM were mixed and incubated at room temperature for 30 mins. Fluorescence was measured as described in Figure 5C.

To confirm that rVRF1 metalloprotease has enzymatic activity, we designed a suitable substrate. It is well documented that metalloproteases cleave the Cys-Glu peptide bond within the Rel homology domain. Multiple sequence alignment of the N-terminal sequences of *H. armigera* Dorsal (Ha_Dorsal) and other NF-κB factors revealed that the cleavage site Cys^73^/Glu^74^ is conserved (Figure 5A). Thus, a peptide ABCYL-Cys-Glu-Gly-Arg-Ser-Ala-EDANS-NH2 (CEGR) containing the putative cleavage site was synthesized and used as substrate in a fluorimetric assay to measure the enzymatic activity of rVRF1.

When the substrate CEGR was incubated with *H. armigera* hemolymph no fluorescence was observed, thus demonstrating that substrate was not cleaved by host enzymes. rVRF1 show enzyme activity in a fluorimetric assay. When 5 mM ZnCl_2_ or MgCl_2_ was added, the fluorescence value increased more than 8-fold compared with the group that did not have metal ions. However, in the presence of CaCl_2_, the fluorescence value revealed no significant differences. In contrast, addition of the host hemolymph into the reaction resulted in a 40-fold increase in fluorescence (Figure 5B). These results indicated that rVRF1 is Zn^2+^-dependent or Mg^2+^-dependent. More importantly, some proteases in the host hemolymph can activate rVRF1, rendering stronger enzymatic activity.

Furthermore, we incubated different molar ratios of rVRF1 with 0.05 μg/μL substrate CEGR and 5 μL whole hemolymph. The results showed that at low concentrations of rVRF1 (0–5 μM), the fluorescence value corresponded to the concentration of the recombinant protein. However, as the concentration of rVRF1 continued to rise the increase in fluorescence intensity was slow. The kinetic parameter *Km* value of rVRF1 was 4.28 ± 0.5 μM. At the concentration of 20 μM rVRF1, the fluorescence intensity reached a plateau and did not continue to increase further (Figure 5C).

Using 1,10-phenanthroline as a general inhibitor of metalloprotease, we determined the rVRF1 inhibition curve. All reactions contained 0.05 μg/μL of the substrate CEGR, 5 μM rVRF1, 5 μL whole hemolymph of *H. armigera*, and increasing amounts of 1,10-phenanthroline. As the inhibitor concentration increased (0.01–0.1 mM), the fluorescence intensity decreased. Thus, we found that 0.18 mM 1,10-phenanthroline could effectively inhibit the activity of rVRF1 (Figure 5D). Taken together, these results showed that 1,10-phenanthroline could effectively inhibit the rVRF1 enzymatic activity, and that rVRF1 had high enzymatic activity after activation by the host hemolymph.

### Interaction between *M. mediator* VRF1 and *H. armigera* dorsal

**Figure 6:**
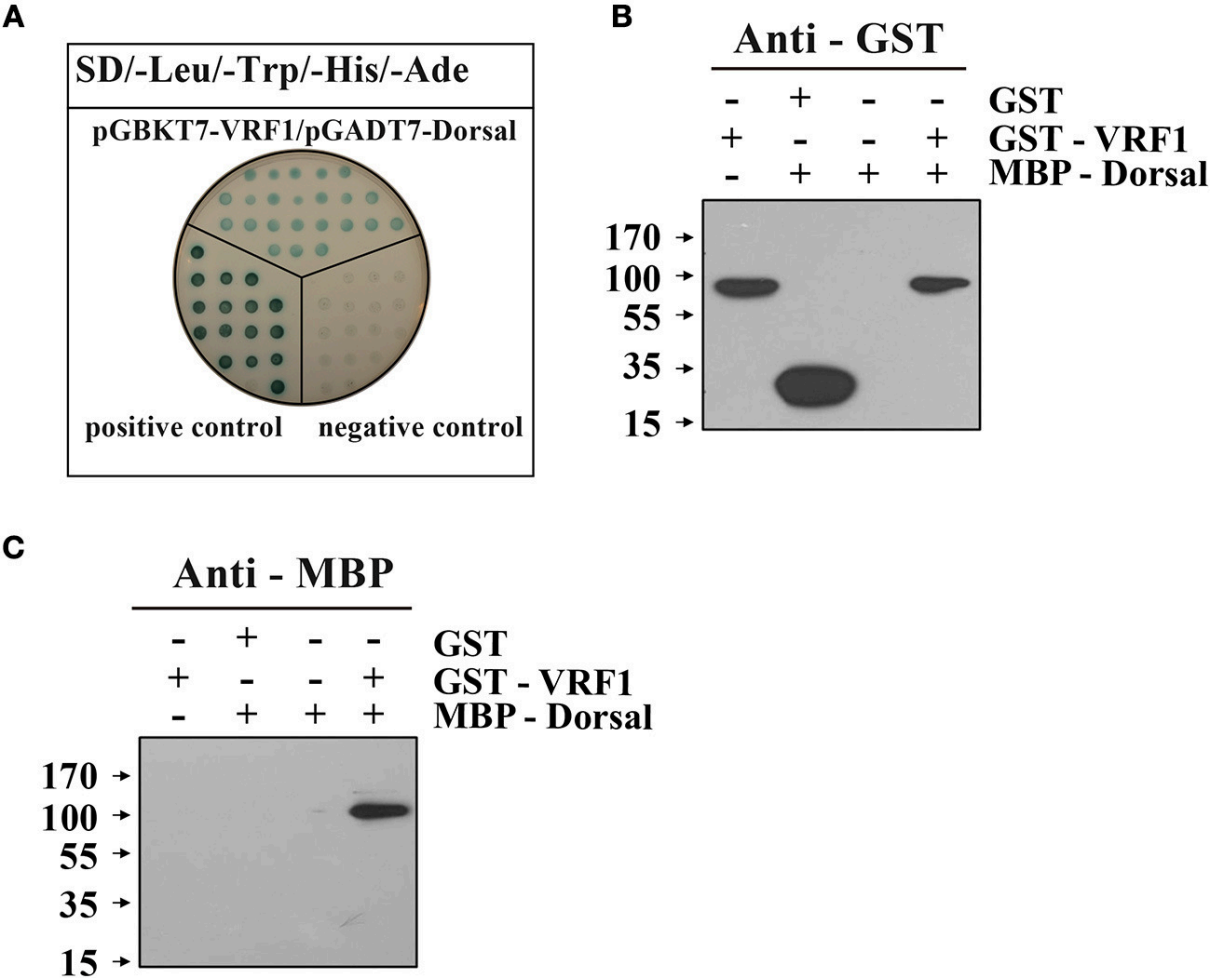
*M. mediator* VRF1 binds *H. armigera* Dorsal. **(A)** Yeast two hybrid assay between *M. mediator* VRF1 and *H. armigera* Dorsal in high-stringency binding test media of SD-Leu-Trp-His-Ade with X-α-gal. pGBKT7-53 and pGADT7-T were cotransformed as positive controls, while pGBKT7-Lam and pGADT7-T were cotransformed as negative controls. **(B)** *In vitro* GST-pull-down assay of recombinant MBP-Dorsal and GST-VRF1. Immunoblot analysis of eluted proteins from Glutathione Sepharose 4B using GST antibody. **(C)** Immunoblot analysis of eluted proteins from Glutathione Sepharose 4B using MBP antibody.

To examine the interaction between VRF1 and *H*. *armigera* Dorsal *in vitro*, we performed a yeast two-hybrid assay. In a high-stringency selective growth medium, co-transformation of pGBKT7-VRF1 and pGADT7-Dorsal rescues the yeast growth (Figure 6A). The yeast two-hybrid analysis identified a host target of VRF1 and linked its metalloprotease activity through its physical interactions. It was also confirmed using a pull-down assay. The full-length coding region of *M*. *mediator VRF1* without signal peptide was cloned into pGEX-4T-1, and the full-length coding sequence of *H*. *armigera Dorsal* was cloned into pMAL-c5x. VRF1 and Dorsal were expressed in *E. coli* cells as fusion proteins with glutathione S-transferase (GST) or maltose-binding protein (MBP) tags, respectively (Supplementary Figure 5). GST-VRF1 was able to pull down MBP-Dorsal, but GST alone did not pull down Dorsal proteins as observed on immunoblots (Figures 6B,C). These results indicated that VRF1 interacts directly with Dorsal.

**Figure 7:**
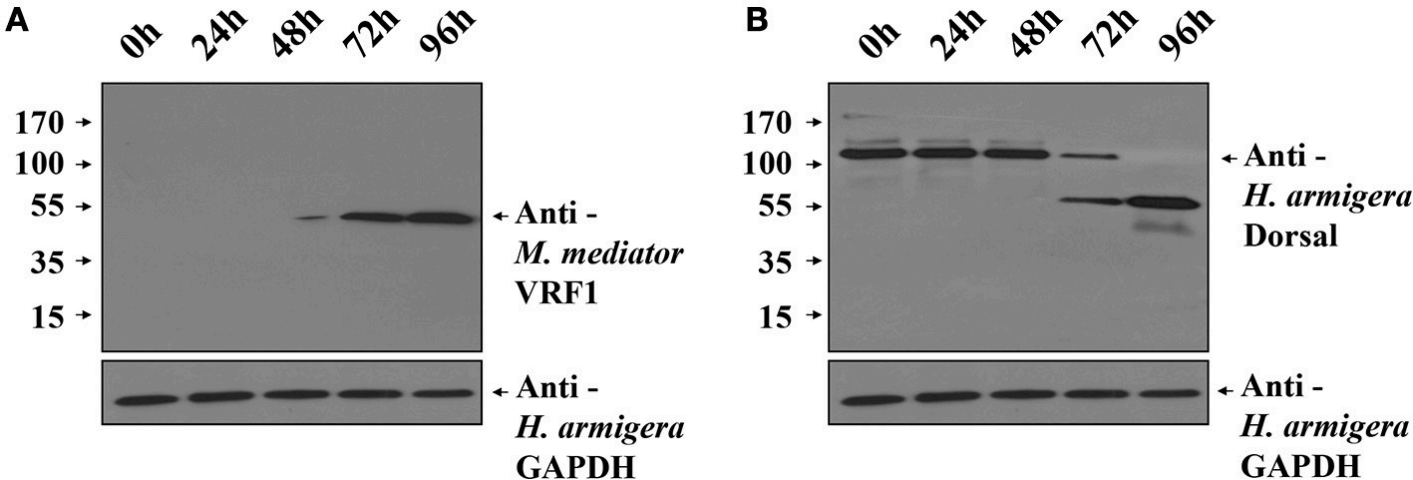
Overexpression of *M. mediator* VRF1 in an *H. armigera* cell line. **(A)** Immunoblot analysis of IOZCAS-Ha-I cells after transfection with pIZT/VRF1^151−483^ post 0, 24, 48, 72, and 96 h using *M. mediator* VRF1 antibody. GAPDH (*H. armigera* glyceraldehyde-3-phosphate dehydrogenase) antibody (1:5,000) was used as the loading control for cells. **(B)** Immunoblot analysis of IOZCAS-Ha-I cells after transfection with pIZT/VRF1^151−483^ post 0, 24, 48, 72, and 96 h using *H. armigera* Dorsal antibody. GAPDH (*H. armigera* glyceraldehyde-3-phosphate dehydrogenase) antibody (1:5,000) was used as the loading control for cells.

To investigate the function of *M*. *mediator* VRF1 *in vivo*, we used *H. armigera* cell line (IOZCAS-Ha-I cells) to overexpress VRF1. Our study suggested that C-terminal (VRF1^151−483^) fragment of *M*. *mediator* VRF1 contained catalytic domain and appeared in the host hemocytes at 6 h post-parasitism. Therefore, we transfected IOZCAS-Ha-I cells with 2 μg of pIZT/VRF1^151−483^. After 72 h, *M*. *mediator* VRF1^151−483^ was strongly expressed in the transfected cells and appeared as a single band at 45 kDa (Figure 7A). Furthermore, at 72 h, *H. armigera* Dorsal was cleaved into two major fragments and at 96 h, *H. armigera* Dorsal was completely cleaved (Figure 7B). We also performed immunoblot analysis of hemocytes at 24 h post-parasitism. The results showed that lower band of Dorsal (~60 kDa) appeared in the second instar *H*. *armigera* larvae hemocytes 24 h post-parasitism (P 24h) but was not observed in unparasitized (N 24h) host hemocytes (Supplementary Figure 6). Based on these results, it was clear that *M*. *mediator* VRF1 mediates *H. armigera* Dorsal cleavage both *in vitro* and *in vivo*.

### VRF1 interferes with the toll/dorsal signaling pathway in host hemocytes

In *H*. *armigera*, Dorsal is postulated to induce the expression of antimicrobial peptides (AMPs), Toll/Dorsal pathway responded to wasp parasitism in some host insects, especially in the regulation of encapsulation (7–11, 33). In order to assess whether VRF1 affected the Toll signaling pathway in *H*. *armigera*, second instar larvae were treated with BSA, venom of *M. mediator*, rVRF1, or both venom and rVRF1. It was found that the survival rate was very low (<20%) after injecting high doses of venom or rVRF1. Therefore, we reduced the injection concentration of the venom (0.05 w.e. venom/larva) and rVRF1 (2 × 10^−7^ μg rVRF1/larva). Then, *B. bassiana* (2 × 10^3^ conidia/larva) were used to challenge *H. armigera* 24 h post-treatment.

**Figure 8:**
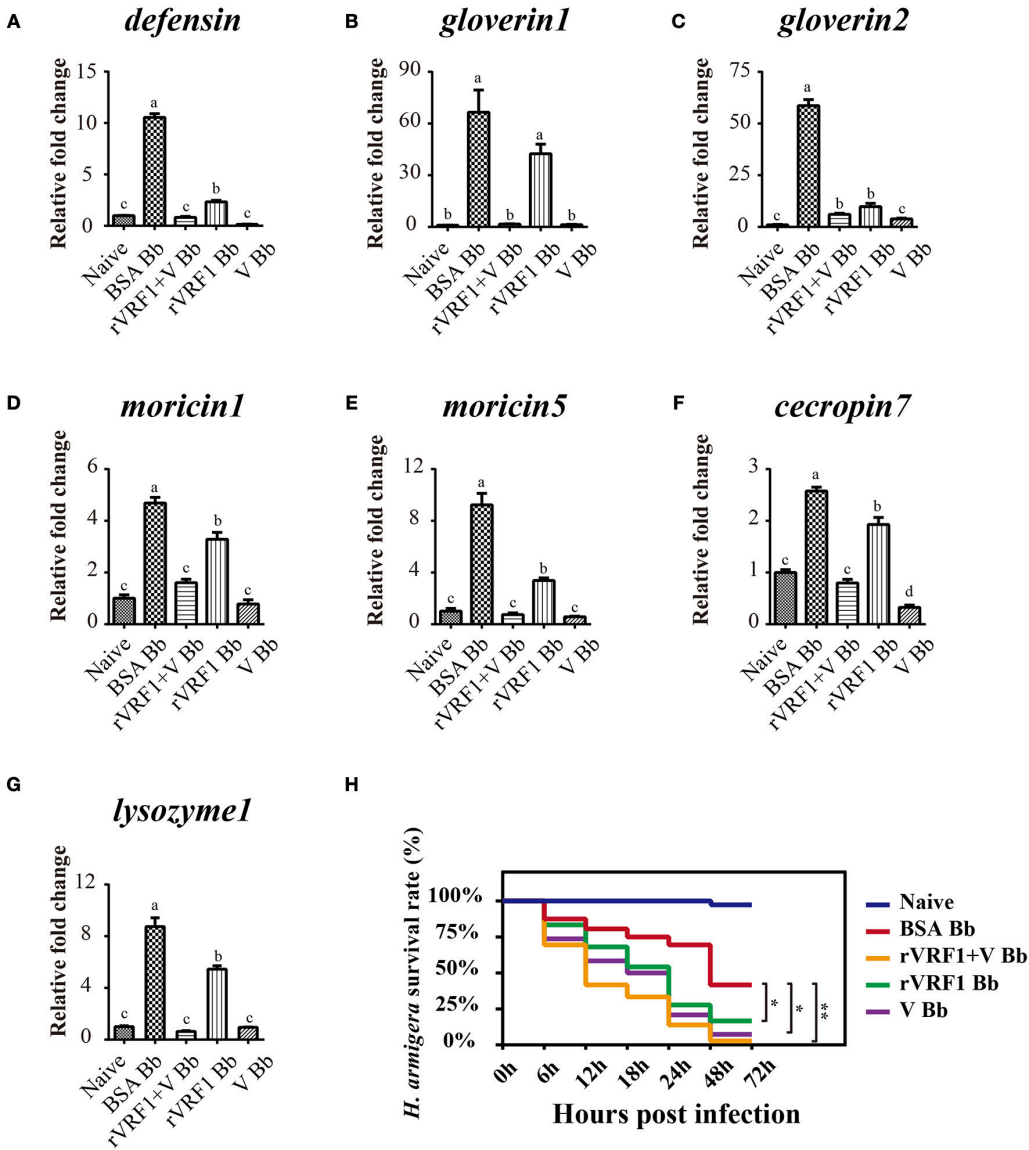
The expression of host AMP genes and survival rate analysis by injection of rVRF1 and venom. **(A–G)** The mRNA expression level of several AMP genes were measured with hemocytes collected from second instar *H. armigera* larvae in the different treatment groups. **(A)** defensin; **(B)** gloverin1; **(C)** gloverin2; **(D)** moricin1; **(E)** moricin5; **(F)** cecropin7; **(G)** lysozyme1. Naïve, untreated second instar larvae of *H. armigera*. BSA Bb, second instar *H. armigera* larvae injected 200 nL of BSA (2× 10^−7^ μg BSA/larva), and one day latter infected with *B. bassiana* (2 × 10^3^ conidia/larva). rVRF1 Bb, larvae were injected 200 nL of rVRF1 (2× 10^−7^ μg rVRF1/larva), and 1 day latter, was infected with *B. bassiana* (2 × 10^3^ conidia/larva). V Bb, larvae were injected 200 nL of venom (0.05 w.e. venom/larva), and 1 day latter infected with *B. bassiana* (2 × 10^3^ conidia/larva). rVRF1+V Bb, larvae were injected 200 nL of 2 × 10^−7^ μg rVRF1 and 0.05 w.e. venom/larva, and 1 day latter challenged with *B. bassiana* (2 × 10^3^ conidia/larva). Error bars represent the means ± SEM from four replicates. Differences between treatments were compared by one-way ANOVA followed by Tukey's test for multiple comparisons. Different letters above a given bar represent significant difference among the different injection groups (*p* < 0.05). **(H)** Survival rate analysis of *H. armigera* after injection and infection experiments. Each group contained 72 s instar larvae and the experiment had three replications. Data were analyzed using the Kaplan-Meier method (^*^p < 0.05, ^**^p < 0.01).

As expected, in the *B. bassiana* infected groups, mRNA expression of the seven AMPs increased markedly due to activation of the Toll pathway. In the rVRF1+V Bb and V Bb group, mRNA abundance of most AMPs did not increase. Furthermore, the expression of several AMPs (except *gloverin1*) was significantly lower in the rVRF1 Bb group compared to the BSA Bb group (Figures 8A–G). We further tested the effects of total venom and rVRF1 on host's resistance to fungal infection. The survival rates in venom-injected group (V Bb) and rVRF1-injected group (rVRF1 Bb) were significantly lower than in the BSA-injected group (BSA Bb) at 48 h post-infection. The venom plus rVRF1-injected group (rVRF1+V Bb) showed the lowest survival rate with fungal infection (Figure 8H). These results suggested that after injecting venom or rVRF1, Toll/Dorsal signaling cascades in host hemocytes were disrupted, and that the synthesis of multiple AMPs was suppressed. Consequently, the suppression of AMPs resulted in increased mortality rate after *B. bassiana* infection. Thus, the toxicity of venom is largely attributed to VRF1.

## Discussion

**Figure 9:**
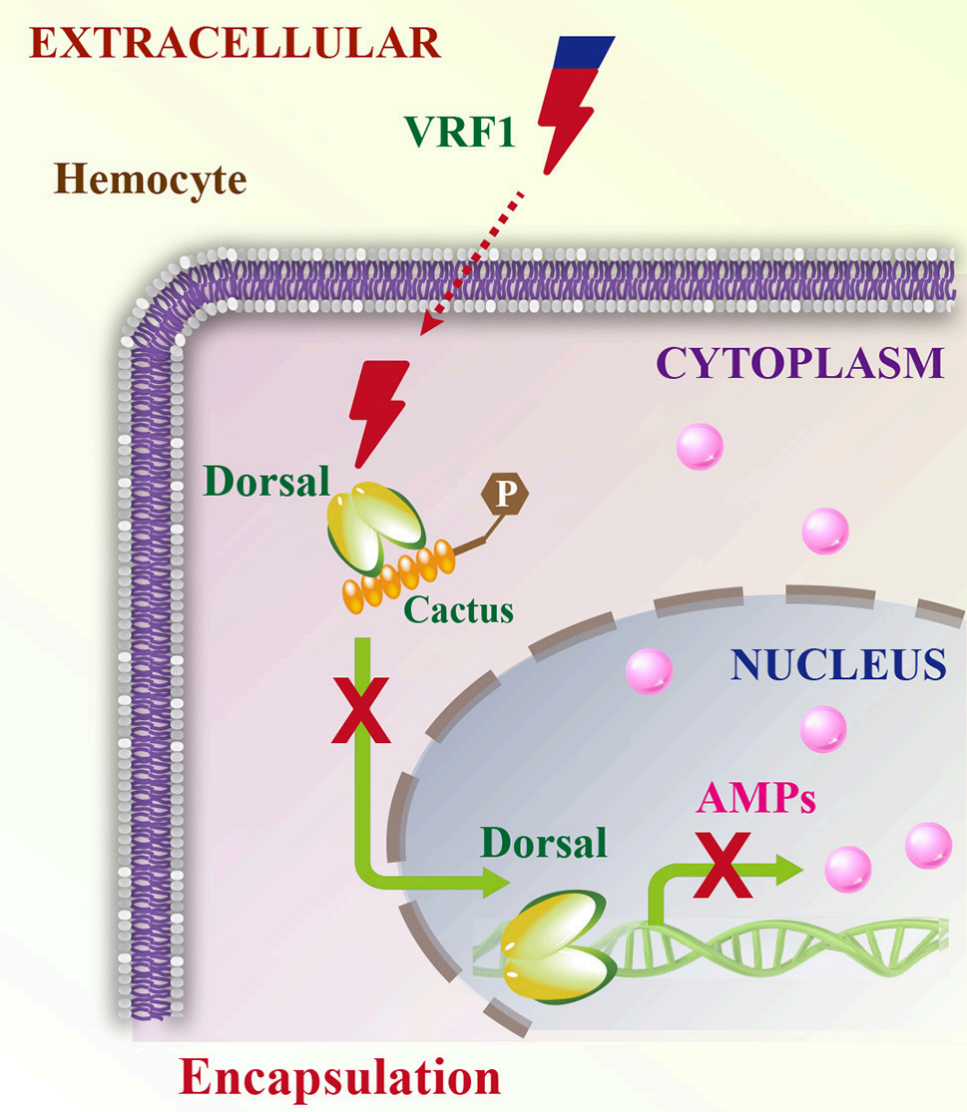
Schematic model of VRF1 action. Venom protein VRF1 (cleaved) from *M. mediator* interacts with Dorsal to suppress the transcription of host cell AMP genes.

Parasitoid wasps are among the most fascinating groups of insects. They are valuable and augmentative biological agents for controlling various insect pests. Mechanisms involved in the suppression of the insect immune responses are complex and delicate, particularly for the koinobiont endoparasitoid wasps, which allow further development of their hosts after parasitism. They modulate the host immune system and ensure the development of their offspring (2, 3). The virulence factors from the koinobiont endoparasitoid wasps are more precise and accurately attack the host without killing it, while ensuring the normal growth of the wasp's offspring. The parasitoid wasp, *M. mediator* is an important biological agent for controlling *H. armigera*. In this study, we report a novel mechanism in which VRF1, a metalloprotease-homolog venom protein, suppresses the Toll/Dorsal immune-signaling pathway in host hemocytes by cleaving Dorsal and interfering with wasp egg encapsulation (Figure 9).

Metalloproteases from parasitoid wasp venoms have long been studied. They were first reported in the venoms of the endoparasitoid wasp *P. hypochondriaca*, were shown to have similarity to reprolysin-type metalloproteases, and were designated as rep1 (35). Although venom from *P. hypochondriaca* can disrupt encapsulation responses, there is no report on the connection between rep1 and egg encapsulation. Later three reprolysin-like metalloproteases (EpMP1-3) were identified from the venom of the ectoparasitic wasp, *E. pennicornis*. EpMP3 is expressed specifically in venom glands and injecting recombinant EpMP3 into fifth instar larvae of the hosts induced mortality and blocked metamorphosis (37). Reprolysin-type metalloproteases belong to the M12B subfamily. Although M12B proteinases from parasitoid wasp venoms have been previously reported, their functions were unknown (23, 35–41). We found that *M. mediator* VRF1 was expressed robustly in venoms, and that it could modulate the wasp egg encapsulation rate in *H. armigera*. In immunoblots, VRF1 appeared as clear protein bands only using 0.2 venom reservoir equivalents or host hemolymph after parasitism. The proenzyme was cleaved in the host hemocoel 6 h post-parasitism and entered the host hemocytes. VRF1 was localized near the host hemocyte nucleus after parasitism and could be distinctly detected even after 24 h using confocal microscopy and immunoblotting. These results indicated that VRF1 can enter the host hemocytes as an extrinsic protein. Some bacterial and animal toxins are capable of traversing the plasma membrane directly (65, 66). The calmodulin-activated adenylate cyclase toxin (CyaA) produced by *Bordetella pertussis* is able to deliver its amino-terminal part into the target cells (67). Alternatively, Several Gram-negative pathogens deliver effector proteins using a needle-like structure called type III secretion system (T3SS) (68). Apart from that, numerous proteins utilize endocytic pathways, which include clathrin-mediated endocytosis (CME), caveolae, macropinocytosis, and phagocytosis (69–71). The CME is the major endocytic mechanism and includes the assembly and maturation of clathrin-coated pits (CCPs), which concentrate cargo proteins and release clathrin-coated vesicles (CCVs). It is strictly mediated by receptors on the cell surface (72).

Although the mechanism of VRF1 entry into the host cells is unclear, several studies have shown that as virulence factors in microorganisms, metalloproteases can cleave NF-κB factors in host cells to disrupt immune-signaling pathways through a conserved type III secretion system (73). NleC, a metalloprotease secreted by enteropathogenic *E. coli* and *Citrobacter rodentium*, repressed host NF-κB-dependent transcription by cleaving NF-κB factor p65 between Cys^38^ and Glu^39^ (48–52, 54). Similarly, metalloprotease PipA, an effector protein in the enteropathogenic bacteria, *Salmonella typhimurium*, directly targeted host NF-κB factor p65 (55). AIP56 is a metalloprotease capable of acting at a distance. It is a major virulence factor of *Photobacterium damselae piscicida* and cleaves sea bass p65 between Cys^39^ and Glu^40^ (47, 53). NF-κB family proteins are evolutionarily conserved and contain a DNA-binding/dimerization domain called RHD. The mammalian Rel proteins consist of p65 (RelA), RelB, c-Rel, the compound proteins p50/p105 (NF-κB1) and p52/p100 (NF-κB2). All NF-κBs form dimers and are sequestered by inhibitors of κB (IκB). IκBs contain ankyrin repeats, which could mask the nuclear localization signals (74–77). For example, Ank-H4 and Ank-N5 from MdBV, and *CsIV-P-vank-1* from CsIV, are homologs of IκB proteins (32, 33). They are encoded by polydnaviruses (PDVs) that bind and disrupt the NF-κB signaling pathway. However, PDVs are symbiotic viruses not present in all parasitoid wasps (78). The venom apparatus is an indispensable organ in all parasitoid wasps and contains a complex mixture of proteins. Our findings provide insight into another virulence mechanism that represses the host immune responses.

It is known that *H. armigera* Dorsal is homologous to p65 and has Cys^73^ and Glu^74^ peptide bonds similar to the p65 cleavage site. We showed that rVRF1 has high enzymatic activity on the synthesized substrate CEGR peptide containing the cleavage site in the presence of the host hemolymph. The Cys^38^ and Glu^39^ peptide bond binds to the phosphate backbone of human p65 binding (74). We also showed the interactions between VRF1 and *H. armigera* Dorsal using yeast two-hybrid and pull-down assays. *M. mediator* VRF1 might have other functions like IκB to bind Dorsal directly. We presume that *M*. *mediator* VRF1, as an abundant factor with high virulence might have other targets in the host cells, some of which could be better substrates than Dorsal. In *H*. *armigera*, Toll and IMD are two of the essential immune response signaling pathways that mediate the induction of AMPs via NF-κB transcription factors (17–19). After injection of venom and rVRF1, we used *B. bassiana* to infect *H. armigera* and found that multiple AMPs were suppressed. However, inhibition of AMPs production is more complicated after parasitism. In *Drosophila*, the specific hematopoietic response to parasitism occurred in the hematopoietic organs, such as the lymph gland and the posterior-signaling center (11). We postulate that VRF1 interferes with NF-κB immune signaling pathways, accurately targeting few, but specific, hemocytes in their host.

In conclusion, our study demonstrated that the wasp venom protein disrupts its host hemocytes' function and inhibits NF-κB function. We present a novel mechanism through which metalloprotease interferes with host immune-signaling cascades (Figure 9). This study also provides insights into developing a promising biological agent for agricultural pest control.

## Author contributions

ZL, ZZ, and ZQL: experimental design; ZL, YC, R-JW, JD, OV, and YH: performed experiments; J-CL and Z-YL: contributed reagents and materials; J-CL and ZZ: writing the original draft; ZL, ZZ, and ZQL: reviewing and editing the manuscript.

### Conflict of interest statement

The authors declare that the research was conducted in the absence of any commercial or financial relationships that could be construed as a potential conflict of interest.

## References

[1] Asgari S Rivers DB. Venom proteins from endoparasitoid wasps and their role in host-parasite interactions. Annu Rev Entomol. (2011) 56:313–35. 10.1146/annurev-ento-120709-144849 20822448

[2] Strand MR Pech LL. Immunological basis for compatibility in parasitoid-host relationships. Annu Rev Entomol. (1995) 40:31–56. 10.1146/annurev.en.40.010195.000335 7810989

[3] Harvey JA Poelman EH Tanaka T. Intrinsic inter- and intraspecific competition in parasitoid wasps. Annu Rev Entomol. (2013) 58:333–51. 10.1146/annurev-ento-120811-153622 23092242

[4] Russo J Dupas S Frey F Carton Y Brehelin M. Insect immunity: early events in the encapsulation process of parasitoid (*Leptopilina boulardi*) eggs in resistant and susceptible strains of *Drosophila*. Parasitology (1996) 112:135–42. 10.1017/S0031182000065173 8587797

[5] Mortimer NT Kacsoh BZ Keebaugh ES Schlenke TA. Mgat1-dependent N-glycosylation of membrane components primes *Drosophila melanogaster* blood cells for the cellular encapsulation response. PLoS Pathog. (2012) 8:e1002819. 10.1371/journal.ppat.1002819 22829770 PMC3400557

[6] Anderl I Vesala L Ihalainen TO Vanha-aho LM Ando I Ramet M. Transdifferentiation and proliferation in two distinct hemocyte lineages in *Drosophila melanogaster* larvae after wasp infection. PLoS Pathog. (2016) 12:e1005746. 10.1371/journal.ppat.1005746 27414410 PMC4945071

[7] Sorrentino RP Melk JP Govind S. Genetic analysis of contributions of dorsal group and JAK-Stat92E pathway genes to larval hemocyte concentration and the egg encapsulation response in *Drosophila*. Genetics (2004) 166:1343–56. 10.1534/genetics.166.3.1343 15082553 PMC1470785

[8] Zettervall CJ Anderl I Williams MJ Palmer R Kurucz E Ando I. A directed screen for genes involved in *Drosophila* blood cell activation. Proc Natl Acad Sci USA. (2004) 101:14192–7. 10.1073/pnas.0403789101 15381778 PMC521135

[9] Matova N Anderson KV Rel/NF-kappa B double mutants reveal that cellular immunity is central to *Drosophila* host defense. Proc Natl Acad Sci USA. (2006) 103:16424–9. 10.1073/pnas.0605721103 17060622 PMC1637598

[10] Schlenke TA Morales J Govind S Clark AG. Contrasting infection strategies in generalist and specialist wasp parasitoids of *Drosophila melanogaster*. PLoS Pathog. (2007) 3:1486–501. 10.1371/journal.ppat.0030158 17967061 PMC2042021

[11] Louradour I Sharma A Morin-Poulard I Letourneau M Vincent A Crozatier M. Reactive oxygen species-dependent Toll/NF-kappaB activation in the *Drosophila* hematopoietic niche confers resistance to wasp parasitism. Elife (2017) 6:e25496. 10.7554/eLife.25496 29091025 PMC5681226

[12] Wu KM Guo YY. The evolution of cotton pest management practices in China. Annu Rev Entomol (2005) 50:31–52. 10.1146/annurev.ento.50.071803.130349 15355239

[13] Slovak M Biological observations on *Microplitis Mediator* Hal (Hym, Braconidae). Biologia (1985) 40:987–96.

[14] Guo J Lu Z Qu Z Pan W Li J. Mass rearing methods and biology of *Microplitis mediator* Haliday (Hymenoptera: Braconidae) in China, a candidate for biological control of *Helicoverpa armigera* (Lepidoptera: Noctuidae). Commun Agric Appl Biol Sci. (2009) 74:393–5. 20222595

[15] Liu XX Zhang QW Zhao JZ Cai QN Xu HL Li JC Effects of the Cry1Ac toxin of *Bacillus thuringiensis* on *Microplitis mediator*, a parasitoid of the cotton bollworm, *Helicoverpa armigera*. Entomol Exp Appl. (2005) 114:205–13. 10.1111/j.1570-7458.2005.00248.x

[16] Li JC Yan FM Coudron TA Pan WL Zhang XF Liu XX Field release of the parasitoid *Microplitis mediator* (Hymenoptera: Braconidae) for control of *Helicoverpa armigera* (Lepidoptera: Noctuidae) in cotton fields in Northwestern China's Xinjiang Province. Environ Entomol. (2006) 35:694–9. 10.1603/0046-225x-35.3.694

[17] Xiong GH Xing LS Lin Z Saha TT Wang CS Jiang HB. High throughput profiling of the cotton bollworm *Helicoverpa armigera* immunotranscriptome during the fungal and bacterial infections. BMC Genomics (2015) 16:321–43. 10.1186/S12864-015-1509-1 26001831 PMC4490664

[18] Pearce SL Clarke DF East PD Elfekih S Gordon KHJ Jermiin LS Genomic innovations, transcriptional plasticity and gene loss underlying the evolution and divergence of two highly polyphagous and invasive *Helicoverpa* pest species. BMC Biol. (2017) 15:63 10.1186/s12915-017-0402-6 28756777 PMC5535293

[19] Xing LS Yuan CF Wang ML Lin Z Shen BC Hu ZH Dynamics of the interaction between cotton bollworm *Helicoverpa armigera* and nucleopolyhedrovirus as revealed by integrated transcriptomic and proteomic analyzes. Mol Cell Proteomics (2017) 16:1009–28. 10.1074/mcp.M116.062547 28404795 PMC5461534

[20] Yuan C Xing L Wang M Wang X Yin M Wang Q. Inhibition of melanization by serpin-5 and serpin-9 promotes baculovirus infection in cotton bollworm *Helicoverpa armigera*. PLoS Pathog. (2017) 13:e1006645. 10.1371/journal.ppat.1006645 28953952 PMC5633200

[21] Cheng Y Lin Z Wang JM Xing LS Xiong GH Zou Z. CTL14, a recognition receptor induced in late stage larvae, modulates anti-fungal immunity in cotton bollworm *Helicoverpa armigera*. Dev Comp Immunol. (2018) 84:142–52. 10.1016/j.dci.2018.02.010 29453998

[22] Burke GR Thomas SA Eum JH Strand MR. Mutualistic polydnaviruses share essential replication gene functions with pathogenic ancestors. PLoS Pathog. (2013) 9:e1003348. 10.1371/journal.ppat.1003348 23671417 PMC3649998

[23] Burke GR Strand MR. Systematic analysis of a wasp parasitism arsenal. Mol Ecol. (2014) 23:890–901. 10.1111/mec.12648 24383716 PMC4120856

[24] Strand MR Burke GR. Polydnaviruses: nature's genetic engineers. Annu Rev Virol. (2014) 1:333–54. 10.1146/annurev-virology-031413-085451 26958725

[25] Bitra K Burke GR Strand MR. Permissiveness of lepidopteran hosts is linked to differential expression of bracovirus genes. Virology (2016) 492:259–72. 10.1016/j.virol.2016.02.023 27011224

[26] Burke GR Simmonds TJ Thomas SA Strand MR. *Microplitis demolitor* bracovirus proviral loci and clustered replication genes exhibit distinct DNA amplification patterns during replication. J Virol. (2015) 89:9511–23. 10.1128/Jvi.01388-15 26157119 PMC4542368

[27] Burke GR. Analysis of genetic variation across the encapsidated genome of *Microplitis demolitor* bracovirus in parasitoid wasps. PLoS ONE (2016) 11:e0158846. 10.1371/journal.pone.0158846 27390861 PMC4938607

[28] Burke GR Walden KKO Whitfield JB Robertson HM Strand MR Genome report: whole genome sequence of the parasitoid wasp *Microplitis demolitor* that harbors an endogenous virus mutualist. G3 (Bethesda) (2018) 8:2875–80. 10.1534/g3.118.200308 30018085 PMC6118312

[29] Burke GR Walden KKO Whitfield JB Robertson HM Strand MR. Widespread genome reorganization of an obligate virus mutualist. PLoS Genet. (2014) 10:e1002722. 10.1371/journal.pgen.1004660 25232843 PMC4169385

[30] Strand MR Burke GR Polydnaviruses: from discovery to current insights. Virology (2015) 479–80:393–402. 10.1016/j.virol.2015.01.018 PMC4424053 25670535

[31] Burke GR Simmonds TJ Sharanowski BJ Geib SM. Rapid viral symbiogenesis via changes in parasitoid wasp genome architecture. Mol Biol Evol. (2018) [Epub ahead of print]. 10.1093/molbev/msy148 30053110

[32] Bitra K Suderman RJ Strand MR. Polydnavirus ank proteins bind NF-kappaB homodimers and inhibit processing of Relish. PLoS Pathog. (2012) 8:e1002722. 10.1371/journal.ppat.1002722 22654665 PMC3359993

[33] Gueguen G Kalamarz ME Ramroop J Uribe J Govind S. Polydnaviral ankyrin proteins aid parasitic wasp survival by coordinate and selective inhibition of hematopoietic and immune NF-kappa B signaling in insect hosts. PLoS Pathog. (2013) 9:e1003580. 10.1371/journal.ppat.1003580 24009508 PMC3757122

[34] Rawlings ND Barrett AJ. Evolutionary families of metallopeptidases. Methods Enzymol. (1995) 248:183–228. 7674922 10.1016/0076-6879(95)48015-3

[35] Parkinson N Conyers C Smith I. A venom protein from the endoparasitoid wasp *Pimpla hypochondriaca* is similar to snake venom reprolysin-type metalloproteases. J Invertebr Pathol. (2002) 79:129–31. 10.1016/S0022-2011(02)00033-2 12095244

[36] Crawford AM Brauning R Smolenski G Ferguson C Barton D Wheeler TT. The constituents of *Microctonus sp*. parasitoid venoms. Insect Mol Biol. (2008) 17:313–24. 10.1111/j.1365-2583.2008.00802.x 18477245

[37] Price DRG Bell HA Hinchliffe G Fitches E Weaver R Gatehouse JA. A venom metalloproteinase from the parasitic wasp *Eulophus pennicornis* is toxic towards its host, tomato moth (*Lacanobia oleracae*). Insect Mol Biol. (2009) 18:195–202. 10.1111/j.1365-2583.2009.00864.x 19320760

[38] De Graaf DC Aerts M Brunain M Desjardins CA Jacobs FJ Werren JH Insights into the venom composition of the ectoparasitoid wasp *Nasonia vitripennis* from bioinformatic and proteomic studies. Insect Mol Biol. (2010) 19(Suppl. 1):11–26. 10.1111/j.1365-2583.2009.00914.x 20167014 PMC3544295

[39] Vincent B Kaeslin M Roth T Heller M Poulain J Cousserans F. The venom composition of the parasitic wasp *Chelonus inanitus* resolved by combined expressed sequence tags analysis and proteomic approach. BMC Genomics (2010) 11:693. 10.1186/1471-2164-11-693 21138570 PMC3091792

[40] Colinet D Deleury E Anselme C Cazes D Poulain J Azema-Dossat C. Extensive inter- and intraspecific venom variation in closely related parasites targeting the same host: the case of *Leptopilina* parasitoids of *Drosophila*. Insect Biochem Mol Biol. (2013) 43:601–11. 10.1016/j.ibmb.2013.03.010 23557852

[41] Doremus T Urbach S Jouan V Cousserans F Ravallec M Demettre E. Venom gland extract is not required for successful parasitism in the polydnavirus-associated endoparasitoid *Hyposoter didymator* (Hym. Ichneumonida*e*) despite the presence of numerous novel and conserved venom proteins. Insect Biochem Mol Biol. (2013) 43:292–307. 10.1016/j.ibmb.2012.12.010 23298679

[42] Colinet D Anselme C Deleury E Mancini D Poulain J Azema-Dossat C. Identification of the main venom protein components of *Aphidius ervi*, a parasitoid wasp of the aphid model *Acyrthosiphon pisum*. BMC Genomics (2014) 15:342. 10.1186/1471-2164-15-342 24884493 PMC4035087

[43] Nishiwaki K Hisamoto N Matsumoto K. A metalloprotease disintegrin that controls cell migration in *Caenorhabditis elegans*. Science (2000) 288:2205–8. 10.1126/science.288.5474.2205 10864868

[44] Mancia F Shapiro L. ADAM and Eph: how ephrin-signaling cells become detached. Cell (2005) 123:185–7. 10.1016/j.cell.2005.10.004 16239135

[45] Takeda S Takeya H Iwanaga S. Snake venom metalloproteinases: structure, function and relevance to the mammalian ADAM/ADAMTS family proteins. Biochim Biophys Acta (2012) 1824:164–76. 10.1016/j.bbapap.2011.04.009 21530690

[46] Giebeler N Zigrino P. A disintegrin and metalloprotease (ADAM): historical overview of their functions. Toxins (2016) 8:122. 10.3390/toxins8040122 27120619 PMC4848645

[47] Silva MT Dos Santos NM do Vale A. AIP56: a novel bacterial apoptogenic toxin. Toxins (2010) 2:905–18. 10.3390/toxins2040905 22069616 PMC3153201

[48] Yen H Ooka T Iguchi A Hayashi T Sugimoto N Tobe T. NleC, a type III secretion protease, compromises NF-kappaB activation by targeting p65/RelA. PLoS Pathog. (2010) 6:e1001231. 10.1371/journal.ppat.1001231 21187904 PMC3002990

[49] Baruch K Gur-Arie L Nadler C Koby S Yerushalmi G Ben-Neriah Y Metalloprotease type III effectors that specifically cleave JNK and NF-kappa B. EMBO J. (2011) 30:221–31. 10.1038/emboj.2010.297 21113130 PMC3020117

[50] Muhlen S Ruchaud-Sparagano MH Kenny B. Proteasome-independent degradation of canonical NFkappaB complex components by the NleC protein of pathogenic *Escherichia coli*. J Biol Chem. (2011) 286:5100–7. 10.1074/jbc.M110.172254 21148319 PMC3037621

[51] Pearson JS Riedmaier P Marches O Frankel G Hartland EL. A type III effector protease NleC from enteropathogenic *Escherichia coli* targets NF-kappaB for degradation. Mol Microbiol. (2011) 80:219–30. 10.1111/j.1365-2958.2011.07568.x 21306441 PMC3178796

[52] Sham HP Shames SR Croxen MA Ma C Chan JM Khan MA. Attaching and effacing bacterial effector NleC suppresses epithelial inflammatory responses by inhibiting NF-kappaB and p38 mitogen-activated protein kinase activation. Infect Immun. (2011) 79:3552–62. 10.1128/IAI.05033-11 21746856 PMC3165486

[53] Silva DS Pereira LM Moreira AR Ferreira-da-Silva F Brito RM Faria TQ. The apoptogenic toxin AIP56 is a metalloprotease A-B toxin that cleaves NF-kappab P65. PLoS Pathog. (2013) 9:e1003128. 10.1371/journal.ppat.1003128 23468618 PMC3585134

[54] Hodgson A Wier EM Fu K Sun X Yu H Zheng W. Metalloprotease NleC suppresses host NF-kappaB/inflammatory responses by cleaving p65 and interfering with the p65/RPS3 interaction. PLoS Pathog. (2015) 11:e1004705. 10.1371/journal.ppat.1004705 25756944 PMC4355070

[55] Sun H Kamanova J Lara-Tejero M Galan JE. A family of *Salmonella* type III secretion effector proteins selectively targets the NF-kappaB signaling pathway to preserve host homeostasis. PLoS Pathog. (2016) 12:e1005484. 10.1371/journal.ppat.1005484 26933955 PMC4775039

[56] Miyoshi S Shinoda S. Microbial metalloproteases and pathogenesis. Microbes Infect. (2000) 2:91–8. 10.1016/S1286-4579(00)00280-X 10717546

[57] Pidde-Queiroz G Magnoli FC Portaro FC Serrano SM Lopes AS Paes Leme AF. P-I snake venom metalloproteinase is able to activate the complement system by direct cleavage of central components of the cascade. PLoS Negl Trop Dis. (2013) 7:e2519. 10.1371/journal.pntd.0002519 24205428 PMC3814341

[58] Duregotti E Zanetti G Scorzeto M Megighian A Montecucco C Pirazzini M Snake and spider toxins induce a rapid recovery of function of botulinum neurotoxin paralyzed neuromuscular junction. Toxins (2015) 7:5322–36. 10.3390/toxins7124887 26670253 PMC4690137

[59] Bustillo S Van de Velde AC Matzner Perfumo V Gay CC Leiva LC. Apoptosis induced by a snake venom metalloproteinase from *Bothrops alternatus* venom in C2C12 muscle cells. Apoptosis (2017) 22:491–501. 10.1007/s10495-017-1350-x 28205127

[60] Chaves-Moreira D Senff-Ribeiro A Wille AC Gremski LH Chaim OM Veiga SS. Highlights in the knowledge of brown spider toxins. J Venom Anim Toxins Incl Trop Dis. (2017) 23:6. 10.1186/s40409-017-0097-8 28194160 PMC5299669

[61] Zhang H Zhang YA Qin Q Wang Y Li X Miao L. A new cell line from larval fat bodies of the bollworm, *Helicoverpa armigera* (Lepidoptera: Noctuidae). In Vitro Cell Dev Biol Anim. (2006) 42:290–3. 10.1290/0605033.1 17316061

[62] Wang RJ Lin Z Jiang H Li JC Saha TT Lu ZY. Comparative analysis of peptidoglycan recognition proteins in endoparasitoid wasp *Microplitis mediator*. Insect Sci. (2015) 24:2–16. 10.1111/1744-7917.12290 26549814

[63] Lu Z Jiang H. Expression of *Manduca sexta* serine proteinase homolog precursors in insect cells and their proteolytic activation. Insect Biochem Mol Biol. (2008) 38:89–98. 10.1016/j.ibmb.2007.09.011 18070668 PMC2199269

[64] Seals DF Courtneidge SA. The ADAMs family of metalloproteases: multidomain proteins with multiple functions. Genes Dev. (2003) 17:7–30. 10.1101/gad.1039703 12514095

[65] Minton NP. Molecular genetics of clostridial neurotoxins. Curr Top Microbiol Immunol. (1995) 195:161–94. 8542753 10.1007/978-3-642-85173-5_8

[66] Sandvig K van Deurs B. Delivery into cells: lessons learned from plant and bacterial toxins. Gene Ther. (2005) 12:865–72. 10.1038/sj.gt.3302525 15815697

[67] Ladant D Ullmann A *Bordetella pertussis* adenylate cyclase: a toxin with multiple talents. Trends Microbiol. (1999) 7:172–6. 10.1016/S0966-842x(99)01468-7 10217833

[68] Galan JE Wolf-Watz H. Protein delivery into eukaryotic cells by type III secretion machines. Nature (2006) 444:567–73. 10.1038/nature05272 17136086

[69] Doherty GJ McMahon HT. Mechanisms of endocytosis. Annu Rev Biochem. (2009) 78:857–902. 10.1146/annurev.biochem.78.081307.110540 19317650

[70] Grant BD Donaldson JG. Pathways and mechanisms of endocytic recycling. Nat Rev Mol Cell Biol. (2009) 10:597–608. 10.1038/nrm2755 19696797 PMC3038567

[71] Wu LG Hamid E Shin W Chiang HC. Exocytosis and endocytosis: modes, functions, and coupling mechanisms. Annu Rev Physiol. (2014) 76:301–31. 10.1146/annurev-physiol-021113-170305 24274740 PMC4880020

[72] Mettlen M Chen PH Srinivasan S Danuser G Schmid SL. Regulation of clathrin-mediated endocytosis. Annu Rev Biochem. (2018) 87:871–96. 10.1146/annurev-biochem-062917-01644 29661000 PMC6383209

[73] Galan JE Lara-Tejero M Marlovits TC Wagner S. Bacterial type III secretion systems: specialized nanomachines for protein delivery into target cells. Annu Rev Microbiol. (2014) 68:415–38. 10.1146/annurev-micro-092412-155725 25002086 PMC4388319

[74] Huxford T Huang DB Malek S Ghosh G. The crystal structure of the IkappaBalpha/NF-kappaB complex reveals mechanisms of NF-kappaB inactivation. Cell (1998) 95:759–70. 9865694 10.1016/s0092-8674(00)81699-2

[75] Jacobs MD Harrison SC. Structure of an IkappaBalpha/NF-kappaB complex. Cell (1998) 95:749–58. 9865693 10.1016/s0092-8674(00)81698-0

[76] Karin M Ben-Neriah Y. Phosphorylation meets ubiquitination: the control of NF-kappa B activity. Annu Rev Immunol. (2000) 18:621–3. 10.1146/annurev.immunol.18.1.621 10837071

[77] Chen ZJ. Ubiquitin signalling in the NF-κB pathway. Nat Cell Biol. (2005) 7:758–65. 10.1038/ncb0805-758 16056267 PMC1551980

[78] Kroemer JA Webb BA. Polydnavirus genes and genomes: emerging gene families and new insights into polydnavirus replication. Annu Rev Entomol. (2004) 49:431–56. 10.1146/annurev.ento.49.072103.120132 14651471

