# Supplementary material for: A Metalloprotease Homolog Venom Protein From a Parasitoid Wasp Suppresses the Toll Pathway in Host Hemocytes
Source: Front Immunol. 2018 Oct 23;9:2301. doi: 10.3389/fimmu.2018.02301 (PMC6206080; doi:10.3389/fimmu.2018.02301)
Supplement: Supplementary file 1 [file Data_Sheet_1.pdf]

Supplementary Material

A metalloprotease homolog venom protein from a parasitoid wasp suppresses the Toll pathway in host hemocytes

Zhe Lin[1*], Yang Cheng[1,4*], Rui-Juan Wang[1,2], Jie Du[1,4], Olga Volovych[1], Jian-Cheng Li[3], Yang Hu[1], Zi-Yun Lu[3], Zhiqiang Lu[†2], and Zhen Zou[†1,4]

*These authors contributed equally to this work.
†To whom correspondence should be addressed.
E-mail: zouzhen@ioz.ac.cn (ZZ); zhiqiang.lu@nwsuaf.edu.cn (ZQL)

Supplementary Figure 1. Cocoon formation rate of three selected metalloproteases in *M. mediator*-injected dsRNA. (A) Cocoon formation rate of offspring measured after depletion of VRF1 (MmV189), **(B)** MmV94, or **(C)** MmV26, when compared with PBS- and dsGFP-injected wasps. Each gene was performed with five replicates, and 20 pupal wasps were included in each treatment. The wasps in every replicate parasitized 24 second instar larvae of *H. armigera* with a single offspring per host. After seven days, we calculated the cocoon formation rate. The percentage of cocoon formation were transformed into a square root of arcsine before analysis using one-way ANOVA. Error bars represent the means ± SEM. Different letters represent the significant difference between groups ($p < 0.05$).

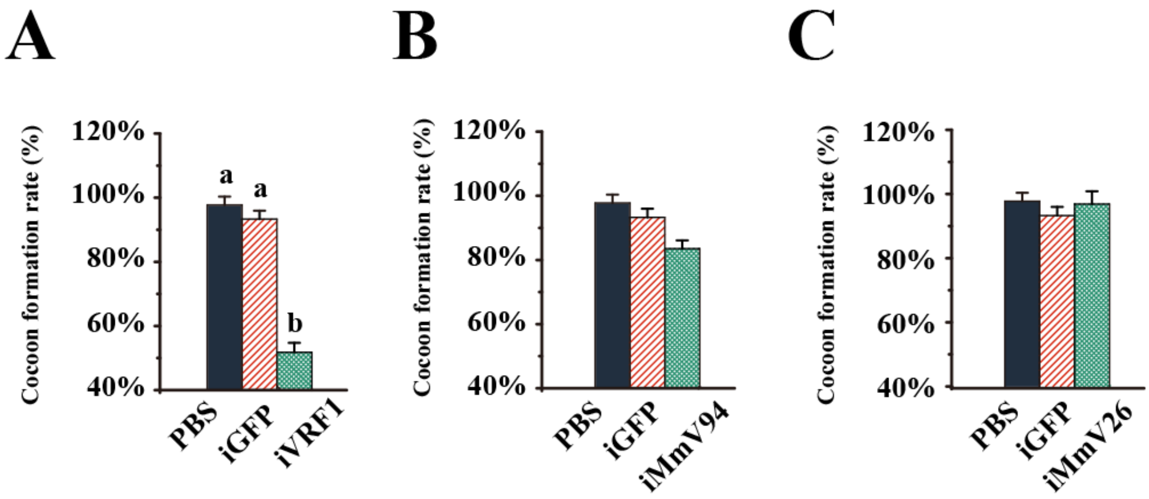

Supplementary Figure 2. Expression and purification of recombinant VRF1. (A) Immunoblot analysis of VRF1 expression in Sf9 cells. Immunoblot was performed by using Anti-His tag monoclonal antibody (CWBio, 1:4000) and HRP-labeled goat anti mouse IgG (Promega, 1:10000). **(B)** SDS-PAGE analysis of purified VRF1 recombinant protein. 1, flow-through buffer; 2-9, elute buffer containing gradient imidazole (50 mM to 1 M).

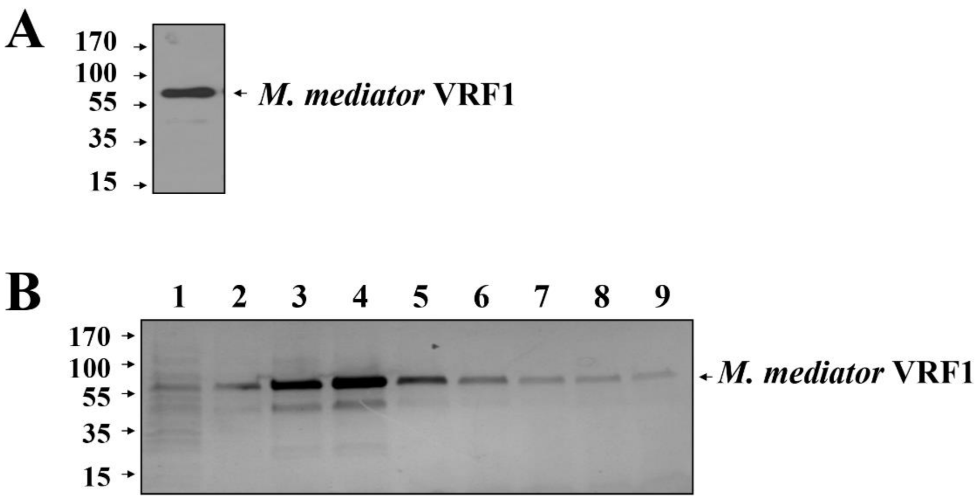

Supplementary Figure 3. Immunoblot analysis of VRF1 in the other two lepidopteran species. Beet armyworm, *Spodoptera exigua*, is an unsuitable host of *M. mediator*, while armyworm, *Pseudaletia separata*, is a suitable host of *M. mediator*. Immunoblot analysis of whole hemolymph from second instar *S. exigua* or *P. separata* larvae at 6 h post-parasitism (P 6h) and unparasitized second instar *S. exigua* or *P. separata* larvae (N 6h), using VRF1 antibody (1:5,000).

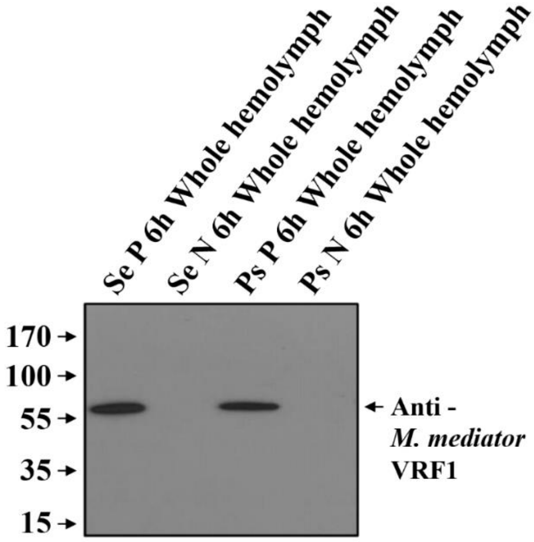

Supplementary Figure 4. Limited proteolysis of rVRF1. (A) Limited proteolysis of rVRF1 with whole hemolymph from second instar *H. armigera* larvae, chymotrypsin, and trypsin. rVRF1 (0.5 μg/μL) was incubated with whole hemolymph, 50 ng/μL of chymotrypsin (Sigma), or 50 ng/μL of trypsin (Sigma) respectively on ice for 30 mins, and mixtures were analyzed by SDS-PAGE. Immunoblot analysis was performed using VRF1 antibody (1:5,000). **(B)** The membrane was stripped with stripping buffer and re-blotted with the 6 × His tag antibody (1:5,000) as described above.

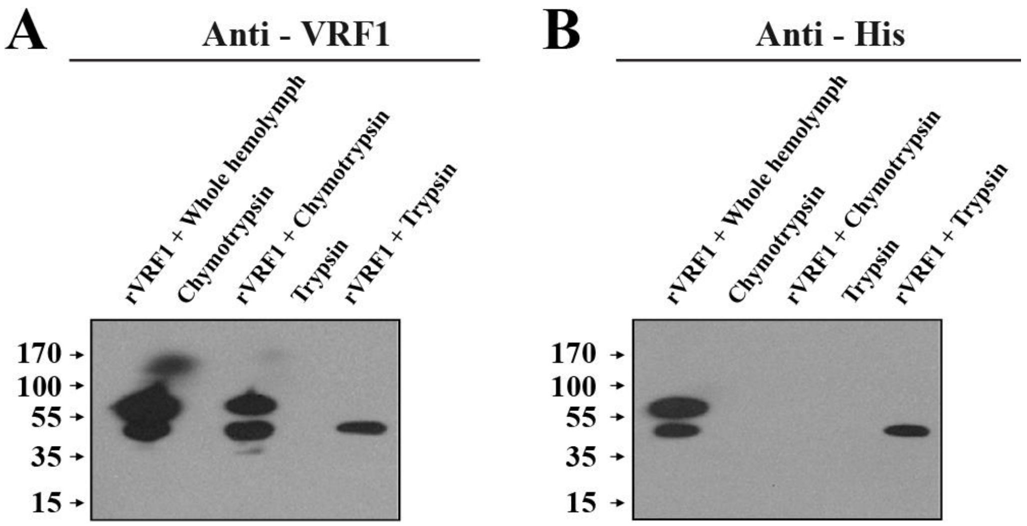

Supplementary Figure 5. Prokaryotic expression of VRF1 and Dorsal. (A) Immunoblot analysis of VRF1 expression in *E. coli* using GST antibody. 1, soluble proteins from *E. coli* transformed with empty pGEX-4T-1 plasmid (28kDa); 2, soluble proteins from *E. coli* transformed with pGEX-4T-1-VRF1 plasmid (84kDa); **(B)** Immunoblot analysis of Dorsal expression in *E. coli* using MBP antibody (43kDa). 3, soluble proteins from *E. coli* transformed with empty pMAL-c5x plasmid; 4, soluble proteins from *E. coli* transformed with pMAL-c5x-Dorsal plasmid (110kDa).

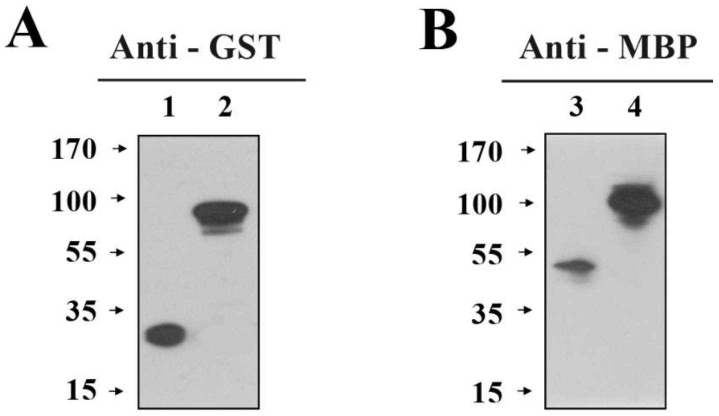

Supplementary Figure 6. Immunoblot analysis of *H. armigera* hemocytes 24 h post-parasitism. Immunoblot analysis of second instar *H. armigera* larvae hemocytes at 24 h post-parasitism, and naive second instar *H. armigera* larvae hemocytes using Dorsal antibody.

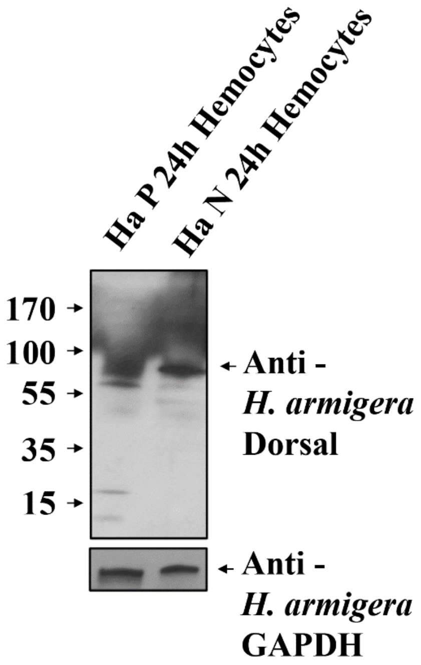

Supplementary Table 1. Sequences of primers used in the study.

Gene	Experiment	Primer sequence (5'-3')	Forward /Reverse
Mm_VRF1	dsRNA	TAATACGACTCACTATAGGGTGGGCAGTAATGTGCGTGAT	F
		TAATACGACTCACTATAGGGTTCAAGCTGCACTGTGACCA	R
MmV94	dsRNA	TAATACGACTCACTATAGGGTGACCAAATGGAATGAAGCAGA	F
		TAATACGACTCACTATAGGGACATTCGGACCAAGTTGGGT	R
MmV26	dsRNA	TAATACGACTCACTATAGGGCCGGCAAGCGGATGTACTAT	F
		TAATACGACTCACTATAGGGGGGCAATGTGACTACCAGCT	R
GFP	dsRNA	TAATACGACTCACTATAGGGCACAAGTTCAGCGTGTCCG	F
		TAATACGACTCACTATAGGGGTTCACCTTGATGCCGTTC	R
Mm_VRF1	5'RACE	CCATCTGTTATTGGGCATCCCGTATTGT	
Mm_VRF1	3'RACE	AAGCCACAACTTCTGAACAACCTCCTCG	
Mm_VRF1	protein expression	CACCATCGGGCGCGGATATGACCGGAGTAATTTTTCTCT	F
		TGATGGCCTTGAAAGTACAAGTTTTCAGCACTTGAGTCATTTATGAG	R
Mm_VRF1	Y2H	ATCTCAGAGGAGGACCTGCATATGGAAGTGATTAATAGAAGACATGTTACA	F
		CGCTGCAGGTCGACGGATTTAAGCACTTGAGTCATTTATGAG	R
Ha_dorsal1	Y2H	CGTACCAGATTACGCTCATATGATGGACATCGGCGGAGAAA	F
		GATTCATCTGCAGCTCGAGCGTAGTCGAGTGCGGGCTGC	R
Mm_VRF1	pull-down	CCGGAATTCCCGGGTCGAGAAGTGATTAATAGAAGACATGTTACA	F

		CGCGAGGCAGATCGTCAGTTAAGCACTTGAGTCATTTATGAG	R
Ha_dorsal1	pull-down	GAGGGAAGGATTTCACATATGGACATCGGCGGAGA	F
		ATGATTACCTGCAGGGAATTCTCAGTAGTCGAGTGCGGG	R
Mm_VRF1	Transfection	CCCGAGCTCGGGATGGTGATATATCCAACGGTTCCGAAG	F
		TCCCCGCGGAGCACTTGAGTCATTTATGAGATT	R
Ha_defensin	qRT-PCR	GAGAGACTCCTCCGTGTTGC	F
		CGTCGTTTCTTCGGAATCGC	R
Ha_gloverin1	qRT-PCR	TCGTGGAGGCTACAAGCAAG	F
		TTCATGCCACTACCACGTCC	R
Ha_gloverin2	qRT-PCR	CAGGGTTACGGGTCCAGGGT	F
		TCCATCGCCAGTGACAGTCA	R
Ha_moricin1	qRT-PCR	TTCGGCTTAGTAGTTCTTGT	F
		GTGGCCAGTGCCGATCGCAC	R
Ha_moricin5	qRT-PCR	AACAACCGACCCCGCTTTC	F
		TTCTTCATGGTGATTCTGGC	R
Ha_cecropin7	qRT-PCR	ACACCATCACGGACGTTGC	F
		GTTCGTGTTCGCGTGTTTGG	R
Ha_lysozyme1	qRT-PCR	GAGTTGAGGAGGCAAGGGTT	F
		ATACAGGCCGTAGTCTCGGG	R
Ha_RpS3	qRT-PCR	ACGGAGTTTTCAAGGCGGAA	F
		GACTGCTCCGGGATGTTGAA	R

Supplementary Data 1. The amino acid sequences of 3 metalloproteases from *M. mediator* and Ha_Dorsal.

>MmV26
MTFKLINKSSKSLWRKRWSELEGGLFVIAIIGFICSILPINASAAVISATSPLGPAHKCTTED
CQVERLELAASIIKTRDTSIKPCDNFFRHVCGNYEDTDIDNNPHIIDTMRETYVDNLIDDD
LATSDSTVNQIIGKIHKVCMDDNARGDKALELLKDVIKKLGGWPILEGDAWKEDDFNWI
DFISNAKKAGYTINYFVDWVPRRQFDGQNHTLQFSLQTAPSFFDYAMSQDSEMQKEMY
KNYITNVATLLGGKLDSATKSLFDLYDFEAKLNDLASDQYSPRTESMTIEELQKEFPSID
WNKLIEKTLTPFLDNDDTKPDLTVFNSKVIEKVIKLMETTPKRVQANYAIWKIVQFTMPF
LSGQFRQADVLFHSVIGYSDMPREEYCDEITKTYTKYAAVNLYLDQFKSSIDTIDKMTALI
KQTMIDMVNDSKNLSDEDKKAAVELLEEMDSTIGQSEKLTDPKELETFYAAAEILDDNF
LHTVLNMNVFKMLTENSNKMRSEIFQYSPMEEVQTDMPENYLNHLYIPAKMIPSTLFDN
TRPMYMNFGAAGSHIAREMFKSLAHLGRKWSEEGEELPGEQIDCFKKIGQNITDTDIKET
LEEIIMDDGIAQYVGFRVAYEAYKQYVIQSGPELGLPELSLTPEQLFWISFSHSLCSAEAD
GPTLPAVIDETLPNLVDDLMLVTLKNVPEISADFDCPVGSRLNPEHKCSWW
>MmV94
MKNGEQEILNDVLTKWNEADKIFEQLGNSKIKLAIAGVVIPTQSDIWRSTDPRKNSSELNE
KYTSSSVLNNITKWLIDNSDRFGSLNYDFFVFVSREQLAYGESFQYVVYVNDFKGSINCDIK
NGSHNRLAGTVVLKTHLVGRYVFGIAEMLGLGTDEKLGCEAGNIMSAKKLNKNPTWSE
CSKREFISFINNSKYSCLHQIPY
>MmV189
MTGVIFLCAVLGVLSQGVHLSEAEVINRRHVTQTKLFGDDMKAWLTPYEGVLATENTPV
YSLVEKPKVSVNDTGYQIIENENAMKNVIAYLREHKSDAITKPSDKPPKANPEQNIVVEA
TTSEQPPRAKTMDDINREIMEMLQKNFPKSRVIYPTVPKTTPKPSVPYPSIVYPEILVIVDN
ALFKTLGSNVRDVLTHVLAFWNGVDLLYRNLESPKFRFNIQAILIIEAPEGFTKLEYAPNK
IDADQVLFTIGGWLYQYQNAFPIDNYDIAVLMTFNHLIERPNNKTLGGIAYSVGACKTSH
ESKRVAKTIAVTETGDFHGIRNMAHELGHSFGIASHDGEGDNTGCPITDGTIMGPSTGNV
GSKYYQWSQCSLNDLTRSFIDGNLVCLYNQICEEGAAVPRLLPGKIADLNKQCDVREAN
TISVINEDNCLEYICRTKENSTGSYEQTTWPVGPADGSSCGEGKMCLLGNCVQENLINDS
SA
>Ha_Dorsal
MDIGGESAIRIGGPQNEEQLNISDVFEAITLADPSFGAGAGAGIDESMARRDQPYVEIVEQ
PASKALRFRYECEGRSAGSIPGVNSTSDNKTYPTIRICGFTGQVVIVVSCVTKDEPYRAHP
HNLVGRERCERGVCTIPTLITKETCEYQFKNLGIQCVKRRDIAEALTVREKLRVDPFRKNF
DHKNHPQSIDLNAVRLCFQVFLPDDSGRLRRPLAPVVSDVIYDKKAMSDLLIMRSSHCSG
TARGGTQVILLCEKVTREDIEVVFYQEENGIVVWEEMAIRILVHKQVAIAFETPAYKYPN
TTDHVHVHFQLKRLSDNARSNSLPFEYIPEFTDVSNKRRKVVSDMLRNYEIDRIYSPEQIK
SEPRDRTPPHHNISSPPLHGYAPPYEQNWNIDNLQGGMAVPGPSHVSPAAAHGNLFVQES
AMWANPQYGQLSPGHMQPGMPQNLQVLQPNMQAMSPSMQPMSPNMQPLSPNMAYGE
RMSPNVAVMSPHGQVMSPHGQALPPHGPGMSPHGPGMQAMSPLGRVSPNVPQNMGHIS

PNLQQQPAYVQQAPQHQAAAGSLMETETHSTTSLSNLLLDRGEHSLLLNSGELAGLSAL
LGDRAQDSQPALDY
